# Mitochondria-associated myosin 19 processively transports mitochondria on actin tracks in living cells

**DOI:** 10.1016/j.jbc.2022.101883

**Published:** 2022-03-31

**Authors:** Osamu Sato, Tsuyoshi Sakai, Young-yeon Choo, Reiko Ikebe, Tomonobu M. Watanabe, Mitsuo Ikebe

**Affiliations:** 1 Department of Cellular and Molecular Biology, University of Texas at Tyler Health Science Center, Tyler, Texas, USA; 2 Laboratory for Comprehensive Bioimaging, RIKEN Center for Biosystems Dynamics Research, Kobe, Hyogo, Japan

**Keywords:** unconventional myosin, intracellular movement, mitochondria, single molecule, TIRF microscopy, CaM, calmodulin, cDNA, complementary DNA, EGFP, enhanced GFP, HEK293T, human embryonic kidney 293T cell line, HILO, highly inclined and laminated optical sheet, HM19, human Myo19, HMF, heavy mitochondrial fraction, LZ, leucine zipper, MSD, mean square displacement, Myo19, myosin 19, NA, numerical aperture, Qdot, quantum dot, R110, rhodamine 110, TIRF, total internal reflection fluorescence

## Abstract

Mitochondria are fundamentally important in cell function, and their malfunction can cause the development of cancer, cardiovascular disease, and neuronal disorders. Myosin 19 (Myo19) shows discrete localization with mitochondria and is thought to play an important role in mitochondrial dynamics and function; however, the function of Myo19 in mitochondrial dynamics at the cellular and molecular levels is poorly understood. Critical missing information is whether Myo19 is a processive motor that is suitable for transportation of mitochondria. Here, we show for the first time that single Myo19 molecules processively move on actin filaments and can transport mitochondria in cells. We demonstrate that Myo19 dimers having a leucine zipper processively moved on cellular actin tracks in demembraned cells with a velocity of 50 to 60 nm/s and a run length of ∼0.4 μm, similar to the movement of isolated mitochondria from Myo19 dimer-transfected cells on actin tracks, suggesting that the Myo19 dimer can transport mitochondria. Furthermore, we show single molecules of Myo19 dimers processively moved on single actin filaments with a large step size of ∼34 nm. Importantly, WT Myo19 single molecules without the leucine zipper processively move in filopodia in living cells similar to Myo19 dimers, whereas deletion of the tail domain abolished such active movement. These results suggest that Myo19 can processively move on actin filaments when two Myo19 monomers form a dimer, presumably as a result of tail–tail association. In conclusion, Myo19 molecules can directly transport mitochondria on actin tracks within living cells.

Myosins are motor proteins that play critical roles in various cell functions, such as force production, cell motility, morphosis, cytokinesis, vesicle, or macromolecule transportation, and organelle localization through association with actin filaments (1). A number of types of myosins have been discovered, and based on the primary structure of their motor domain, it is thought that >35 classes of myosins are constituted of the superfamily (2). All myosins contain a conserved motor domain and neck domain, followed by a tail domain specific to the myosin class. The motor domain is responsible for actin binding and converting the chemical energy of ATP to mechanical work. Following short neck domain containing IQ motifs interacts with calmodulin (CaM) and/or CaM-like light chain(s) and constitutes a lever arm when myosin moves on actin filaments. In addition, this domain also functions as a regulatory domain of various myosins. A C-terminal tail domain is structurally unique and is often critical for the class-specific functions, such as regulation of the motile activity, association with unique binding partners, and targeting the molecule to the specific intracellular structures. Several myosins in the myosin superfamily members have a coiled-coil domain after the neck region to facilitate its dimer formation, whereas others do not have, and it is thought that the dimer formation of myosin is important for the function and regulation of myosin molecules (3, 4, 5, 6).

Recent studies have revealed that several types of myosin can continuously move on actin filament, which are thought to be involved in cargo transportation in cells (7). These myosins are often called processive myosin, and it is thought that these myosins spend a majority of the actin-activated ATP hydrolysis cycle time in the “strong actin-binding” state called high duty cycle myosin (8). It is thought that two-headed myosins having a high duty cycle can processively walk on actin filaments with hand-over-hand mechanism (9).

Myo19 (also called myosin 19 or class XIX myosin) is an unconventional myosin that is expressed in vertebrates. From the primary structure, it is predicted that Myo19 consists of a motor domain, a neck domain containing three IQ motifs, and a unique tail domain, but missing a predicted coiled-coil domain (2, 10). It has been reported that Myo19 is colocalized with mitochondria (11) and has been considered to play a role in actin-based mitochondria dynamics in cells (12, 13, 14, 15, 16, 17). The C-terminal tail domain of Myo19 is necessary and sufficient for mitochondria targeting (11, 13, 14). It is demonstrated that the C-terminal tail domain binds to outer mitochondrial rho GTPase protein (Miro1 and/or Miro2) (17), thus protecting Myo19 from degradation (16). Miro proteins also associate with KIF5 (also called kinesin 1) and dynein–dynactin complex, microtubule-associated motor proteins (18, 19, 20, 21). Therefore, it is anticipated that Miro proteins coordinate both microtubule-based mitochondria movement by KIF5/dynein and actin-based movement by Myo19 in cells. Recent studies have also revealed that Myo19 migrates to the tip of filopodia in cells by stimulations such as glucose starvation and reactive oxygen species (13, 15). This suggests that Myo19 may be regulated by such stimulations.

Biochemical studies have revealed that Myo19 has actin-activated ATPase activity like most other myosins (22, 23, 24) with a plus (barbed)-end-directed (23) high duty ratio motor (23, 24). The IQ domain of Myo19 binds to CaM (a common light chain of unconventional myosins) as well as myosin regulatory light chains (23).

A critical unanswered question is whether Myo19 is a motor protein that is suitable for cargo transportation. As Myo19 exclusively colocalizes with mitochondria, it is anticipated that Myo19 can transport mitochondria if Myo19 has a suitable motor characteristic for cargo transportation (15, 24). Since Myo19 does not have a coiled coil, it has been thought that Myo19 alone is a single-headed motor (2). It is puzzling whether a single-headed Myo19 can move on actin filaments with well-known hand-over-hand mechanism (9). The critical missing information is whether Myo19 can move processively in a way that is suitable for transportation of mitochondria, since accumulation of Myo19 at filopodial tips does not necessarily assure the processive movement of Myo19.

The aim of this study is to clarify whether human Myo19 (HM19) can move processively on actin filaments and transport mitochondria in cells. To clarify the movement of Myo19 and its role in mitochondrial movement, we utilized single-molecule analysis with a highly sensitive total internal reflection fluorescence (TIRF) microscope by highly inclined and laminated optical sheet (HILO) illumination (25, 26, 27).

We characterized the movement of Myo19 at a single-molecule level using demembraned and living cell systems. The movement of single Myo19 molecules was further determined in *in vitro* single-molecule motility assay using single actin filaments by TIRF microscopy. Based upon our findings, we propose that Myo19 is a weak gaiting processive motor, and monomer–dimer transition of Myo19 *via* its tail domain is critical not only for the movement of single Myo19 molecules on actin filaments but also for actin-based mitochondrial movement in cells.

## Results

### HM19 constructs used in this study

**Figure 1:**
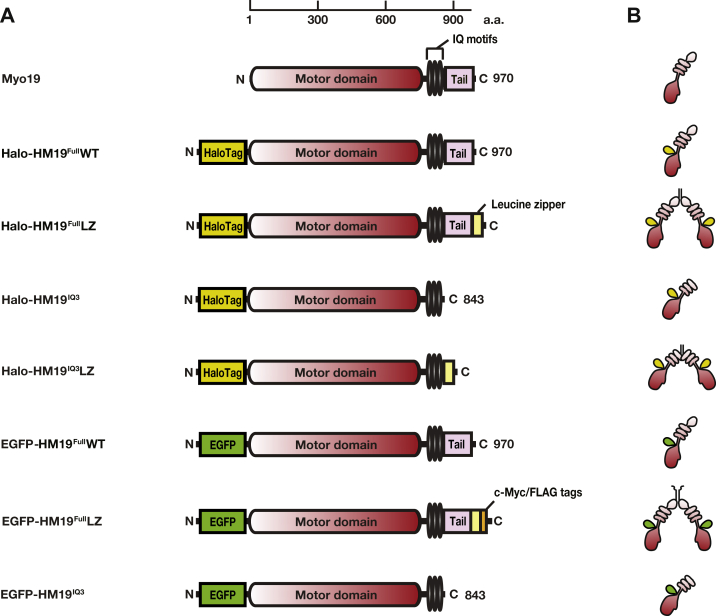
**Schematic drawing of HM19 constructs used in this study.***A*, diagram of domain structures of HM19 constructs. HM19 consists of a motor domain (*dark red*), three IQ motifs (*black*), and a tail domain (*pink*). To observe single-molecule movement of HM19 in living cells, HaloTag was introduced at the N-terminal end (*yellow green*). To produce the dimer form of HM19 construct, a GCN4 leucine-zipper motif was introduced at the C-terminal end of HM19 (*yellow*). To purify the HM19 protein and perform the single-molecule stepping with Qdots, c-Myc/FLAG sequences were introduced at the C-terminal end of the HM19 constructs (*orange*). To confirm the localization of HM19 construct, and single molecular experiments both in cells and *in vitro*, EGFP-HM19 was also made (*green*). The number at the *top of the panel* represents amino acid residues of Myo19. *B*, configuration of HM19 constructs. It is speculated that HM19 dimer is formed when a GCN4 leucine-zipper motif is attached to the C-terminal end of HM19 construct. EGFP, enhanced GFP; HM19, human Myo19; Qdot, quantum dot.

To test the single-molecule movement of Myo19, we made various HM19 constructs (Fig. 1). To visualize HM19 molecules in living cells at a single-molecule level, we employed HaloTag technique. Advantage of this technique is that we can control the number of fluorescently labeled molecules within the cells by manipulating the concentration of exogenously added HaloTag ligand. HaloTag was added to the N-terminal end of HM19 to avoid possible interference of the HaloTag moiety on the interaction of HM19 and its binding partners such as mitochondria or possible influence on interheavy chain dimer formation (Fig. 1). As shown in Figure 1, there is no obvious predicted coiled-coil domain in HM19. It has been shown that mammalian myosin 6, myosin 7a, and myosin 10 do not readily form a dimer, even though they contain a predicted coiled-coil domain (4, 28, 29), and it is thought that dimer formation of these myosins is facilitated by the binding of target molecules (4, 5, 30, 31), and that dimer formation is critical for the continuous movement of these myosins (32, 33, 34). To address the importance of possible dimer formation of HM19 on its movement and cargo transporter activity, we made the forced dimer constructs of HM19-leucine zipper (LZ) (Fig. 1).

It has been reported that the tail domain of myosin can function as an intramolecular inhibitor domain (35, 36, 37). Since the tail domain is involved in the regulation of dimer formation of various myosin family members (29, 30, 38), it is plausible that the tail domain of Myo19 may play a role in dimer formation. In order to study the role of the tail domain in the Myo19 motor function, we also produced a tail-truncated mutant of HM19 construct (Fig. 1).

Although the HaloTag technique assures a single fluorophore in a single HM19 heavy chain, fluorophore-unbound HM19 can be present. In addition to the HaloTag-HM19, therefore, we also produced enhanced GFP (EGFP)-HM19 constructs to observe the single-molecule movement of HM19 (Fig. 1). In order to study the single-molecule stepping of HM19 movement, C-terminal FLAG tag was introduced in aid of protein isolation, and a c-Myc tag was introduced to label a quantum dot (Qdot) at the C-terminal end of the molecule *via* anti-Myc antibodies conjugated with Qdot (Fig. 1). All constructs used in this study were in good agreement in terms of the molecular masses and the response to antibodies (Fig. S1).

### Continuous movement of mitochondria containing HM19 on cellular actin structure

It has been reported that mitochondrial transport can be driven by kinesin–dynein motors (39). On the other hand, it is unclear whether an actin-based motor, myosin such as HM19, can drive mitochondrial movement or transport. We first examined the mitochondrial movements on actin tracks to elucidate the physiological relevance of HM19 in mitochondrial movement in cells.

To address this question, heavy mitochondrial fraction (HMF) was prepared from HM19-untransfected and HM19-transfected human embryonic kidney 293T (HEK293T) cells according to Shneyer *et al.* (13), since HMF contains HM19 (13). The isolated mitochondria vesicles were introduced into the demembraned U2OS cells, and the movement was observed. We first tested the localization of HMF in the absence of ATP to see whether HM19 is associated with cellular actin. After addition of MitoTracker-stained HMF obtained from untransfected HEK293T cells, the demembraned cells were observed (Fig. S2, *A*–*C*). The result clearly showed that the HMF colocalized with actin on demembraned cells, suggesting that mitochondrial membrane vesicles are associated with cellular actin tracks, presumably through HM19 that is known to be associated with mitochondria (11, 13). We also prepared mitochondria vesicles from HEK293 cells expressing Halo-HM19-forced dimer (HM19^Full^LZ) and examined the association of Halo-HM19^Full^LZ with mitochondria and actin tracks. Halo-HM19^Full^LZ was mostly colocalized with mitochondria vesicles (Fig. S2, *D*–*F*). Moreover, Halo-HM19^Full^LZ showed notable colocalization with actin tracks (Fig. S2, *G*–*I*), suggesting the association of Halo-HM19^Full^LZ on mitochondria with actin tracks. Consistently, EGFP-HM19^Full^WT and EGFP-HM19^Full^LZ showed marked colocalization with mitochondria. Moreover, EGFP-HM19^Full^LZ showed notable localization at filopodia (Figs. S3 and S4).

**Figure 2:**
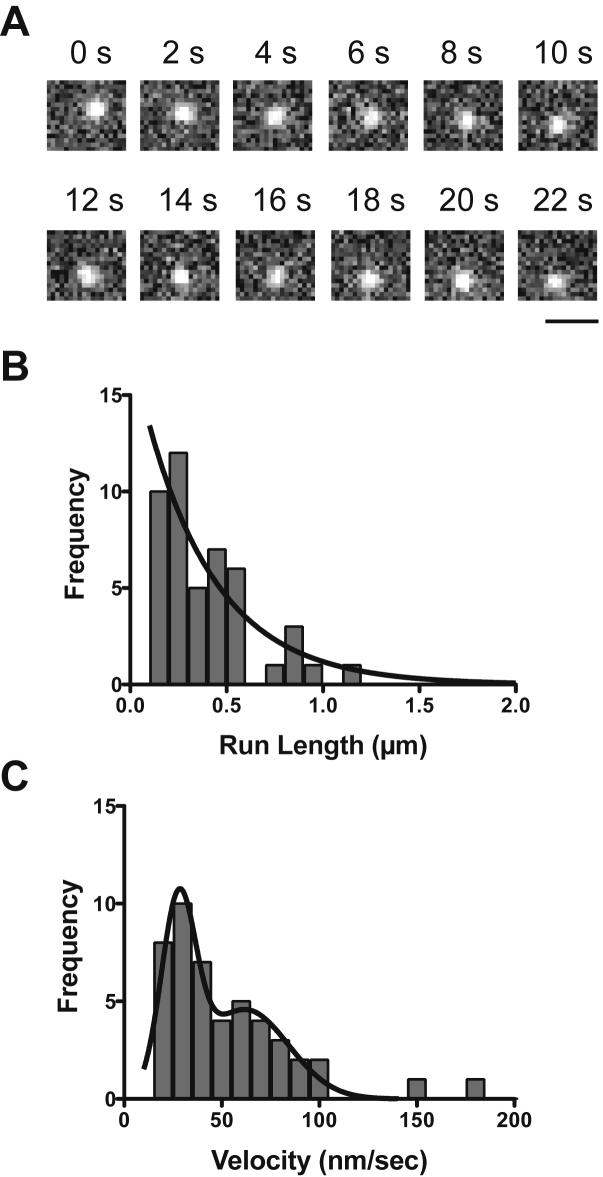
**Movement of HM19**^**Full**^**LZ-associated mitochondria vesicles on demembraned cells.** The movement of Halo-HM19^Full^LZ-associated HMF was observed with a TIRF microscope at 5 fps in the presence of 1 mM ATP, and the run length and velocity were analyzed. *A*, representative movement of HMF prepared from HM19^Full^LZ-expressing cells on demembraned U2OS cells. Halo-HM19^Full^LZ was expressed in HEK293T cells and stained with R110 direct reagent. The HMF prepared as described in the “Experimental procedures” section was added to demembraned U2OS cells, and the movement was observed with a TIRF microscope in the presence of 1 mM ATP (Movie S1). The bar represents 1 μm. *B*, run length of HM19^Full^LZ-associated mitochondria vesicles. The position of the moving light spot was tracked, and the run length was calculated. The *solid line* shows the best fit to a single exponential equation, *R*_*0*_e^−*r*/*λ*^, where *R*_*0*_ is the initial frequency extrapolated to zero run length, *r* is the run length, and *λ* is the average run length. The average run length was 0.37 ± 0.09 μm (SEM, n = 48). *C*, the velocity of HM19^Full^LZ-associated mitochondria vesicles. The distribution was fitted to two Gaussian equations with the mean velocity of 27.8 ± 1.0 and 61.9 ± 6.4 nm/s (SEM, n = 48), respectively. HEK293T, human embryonic kidney 293T cell line; HM19, human Myo19; HMF, heavy mitochondrial fraction; LZ, leucine zipper; R110, rhodamine 110; TIRF, total internal reflection fluorescence.

Next, we examined the movement of mitochondria on demembraned cells using HMF prepared from Halo-HM19^Full^LZ-overexpressed HEK293T cells (see the “Experimental procedures” section). Interestingly, HMF prepared from the Halo-HM19^Full^LZ-expressing cells moved on the cellular actin tracks in the presence of ATP (Fig. 2*A* and Movie S1). The mean run length and the velocity were 0.37 ± 0.09 μm, and 55.7 ± 5.9 nm/s (mean ± SEM, n = 48), respectively (Fig. 2, *B*, *C* and Table 1). The histogram of the velocity showed a wide distribution with the combination of two different peaks at ∼30 and ∼60 nm/s. On the other hand, we did not observe the movement of HMF obtained from HM19-untransfected cells in the presence of ATP. These results support that the HMF movement is driven by HM19.

**Table 1:** Run lengths and velocities of HM19 in demembraned U2OS cells

Examined samples	Run length (*λ*)	Velocity
	μm	nm s^−^^1^
HMF-expressing Halo-HM19^Full^LZ	0.37 ± 0.09	27.8 ±1.0 and 61.9 ± 6.4
	(n = 48)	(n = 48)
Purified Halo-HM19^Full^LZ	0.43 ± 0.08	41.1± 6.4 and 73.3 ± 8.6
	(n = 55)	(n = 55)

Experiments were in the presence of 1 mM ATP (see the “Experimental procedures” section). Mean ± SEM was indicated.

### Processive movement of a forced dimer of HM19 on demembraned cells

**Figure 3:**
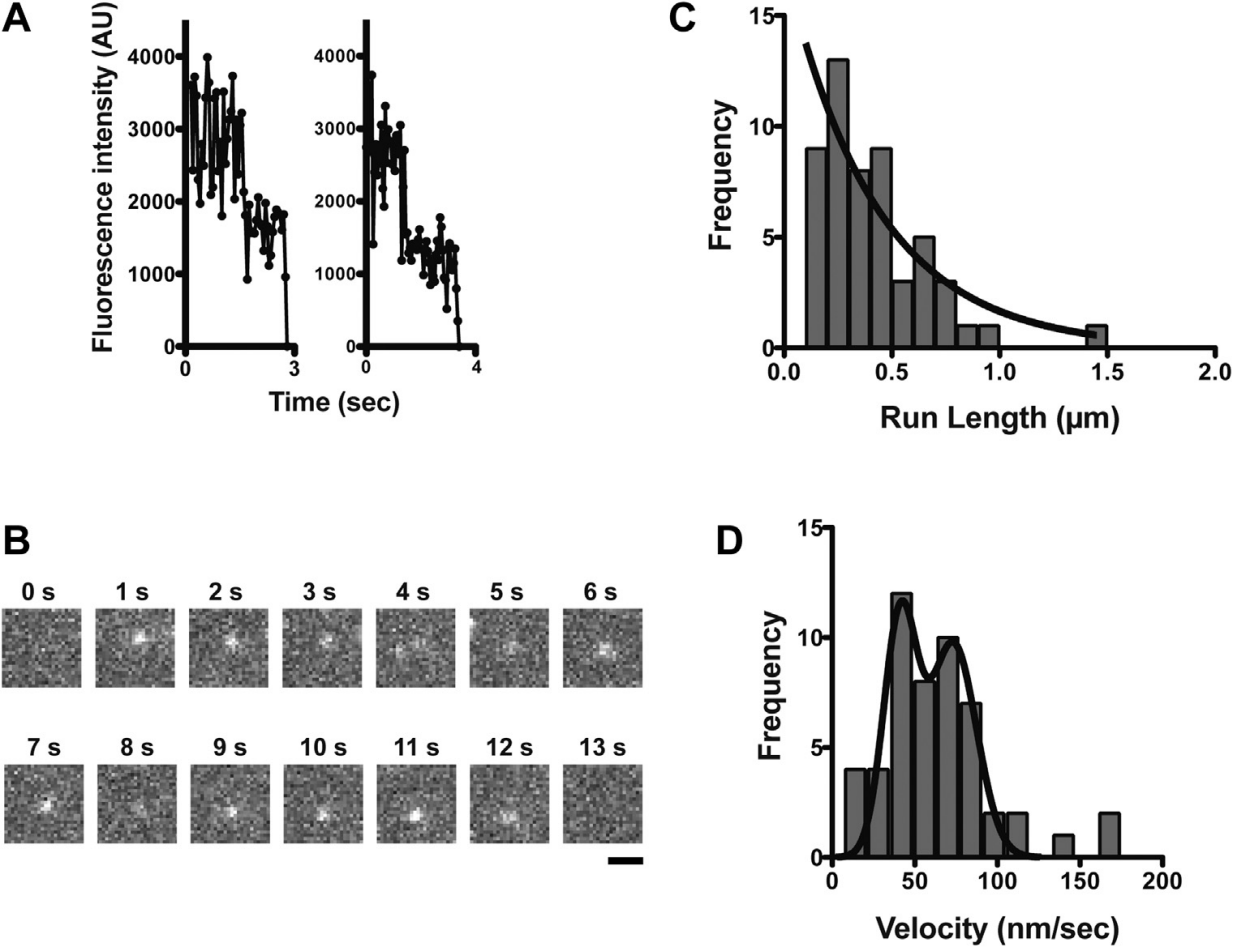
**Single-molecule imaging of purified HM19**^**Full**^**LZ on demembraned cells.** EGFP-HM19^Full^LZ was purified, and the movement on demembraned U2OS cells was observed with a TIRF microscope at 5 fps. *A*, representative two-step photobleaching of the fluorescent spots of EGFP-HM19^Full^LZ. *B*, typical time-lapse images of EGFP-HM19^Full^LZ movement in the presence of 1 mM ATP. EGFP-HM19^Full^LZ single-molecule movement from 0.2 to 12.6 s was recorded. The bar represents 1 μm. The pixel-by-pixel size is 94 nm. *C*, run length of EGFP-HM19^Full^LZ in the presence of 1 mM ATP. The position of moving light spot was tracked, and the run length was calculated. *Solid line* shows the best fit to a single exponential equation, *R*_*0*_e^−*r*/*λ*^. The average run length was 0.43 ± 0.08 μm (SEM, n = 55). *D*, the velocity of EGFP-HM19^Full^LZ in the presence of 1 mM ATP. The distribution was fitted to two Gaussian equations with the mean velocities of 41.1 ± 6.4 and 73.3 ± 8.6 nm/s (SEM), respectively, for EGFP-HM19^Full^LZ. AU, arbitrary unit; EGFP, enhanced GFP; HM19, human Myo19; LZ, leucine zipper; TIRF, total internal reflection fluorescence.

We next asked whether HM19 can move at a single-molecule level on demembraned cells’ actin tracks. For this experiment, we purified EGFP-HM19^Full^LZ from EGFP-HM19^Full^LZ-transfected HEK293T cells by anti-FLAG affinity chromatography, and the movement was observed with a TIRF microscope (see the “Experimental procedures” section). As shown in Figure 3*A*, the fluorescent spots of EGFP signals typically showed two-step photobleaching. This indicates that the fluorescent spots contain two EGFP molecules, which are presumably derived from a single dimer molecule of EGFP-HM19^Full^LZ containing an LZ module. The movement of EGFP-HM19^Full^LZ was monitored under the TIRF microscope in the presence of 1 mM ATP (Movie S2). The single-molecule EGFP-HM19^Full^LZ continuously moved on demembraned cellular actin tracks, and the typical time-lapse images of the movement are shown in Figure 3*B*. The histogram of the run length is shown in Figure 3*C*. The obtained result was best fit to a single exponential equation to yield an average run length of 0.43 ± 0.08 μm (SEM, n = 55; Table 1). The result indicates that EGFP-HM19^Full^LZ moved processively on demembraned-cellular actin in the presence of physiological ATP concentration. Figure 3*D* shows the histogram of the velocity of EGFP-HM19^Full^LZ in 1 mM ATP. The velocity was widely distributed with the peaks at ∼40 nm/s and above ∼70 nm/s (Table 1). These velocity peaks were similar to those obtained for HMF containing Halo-HM19^Full^LZ (Fig. 2*C*) although the population of molecules with faster velocity was larger.

### Processive movement of HM19 in living cells

Colocalization of HM19 constructs with actin or mitochondria was tested by confocal microscopy (Figs. S3–S5). EGFP-HM19 WT (HM19^Full^WT, without LZ) localized at mitochondria but not notably localized at filopodial tips (Fig. S4*B*). On the other hand, EGFP-HM19^Full^LZ localized at both mitochondria and the tip of filopodia (Fig. S4*C*). EGFP-tail-less HM19 (HM19^IQ3^) did not show localization either at mitochondria or at the tip of filopodia (Fig. S4*D*). These results suggest that the tail domain is required for the mitochondrial targeting that is consistent with the previous reports (11, 12, 13, 16, 17). These results also suggest that the dimer formation is important for the movement of HM19 to the filopodial tips. It should be noted that there was no essential difference between EGFP-HM19 constructs and Halo-HM19 constructs in their localizations, consistent with the similar localization of both constructs in living cells and fixed cells (Figs. S3–S5).

**Figure 4:**
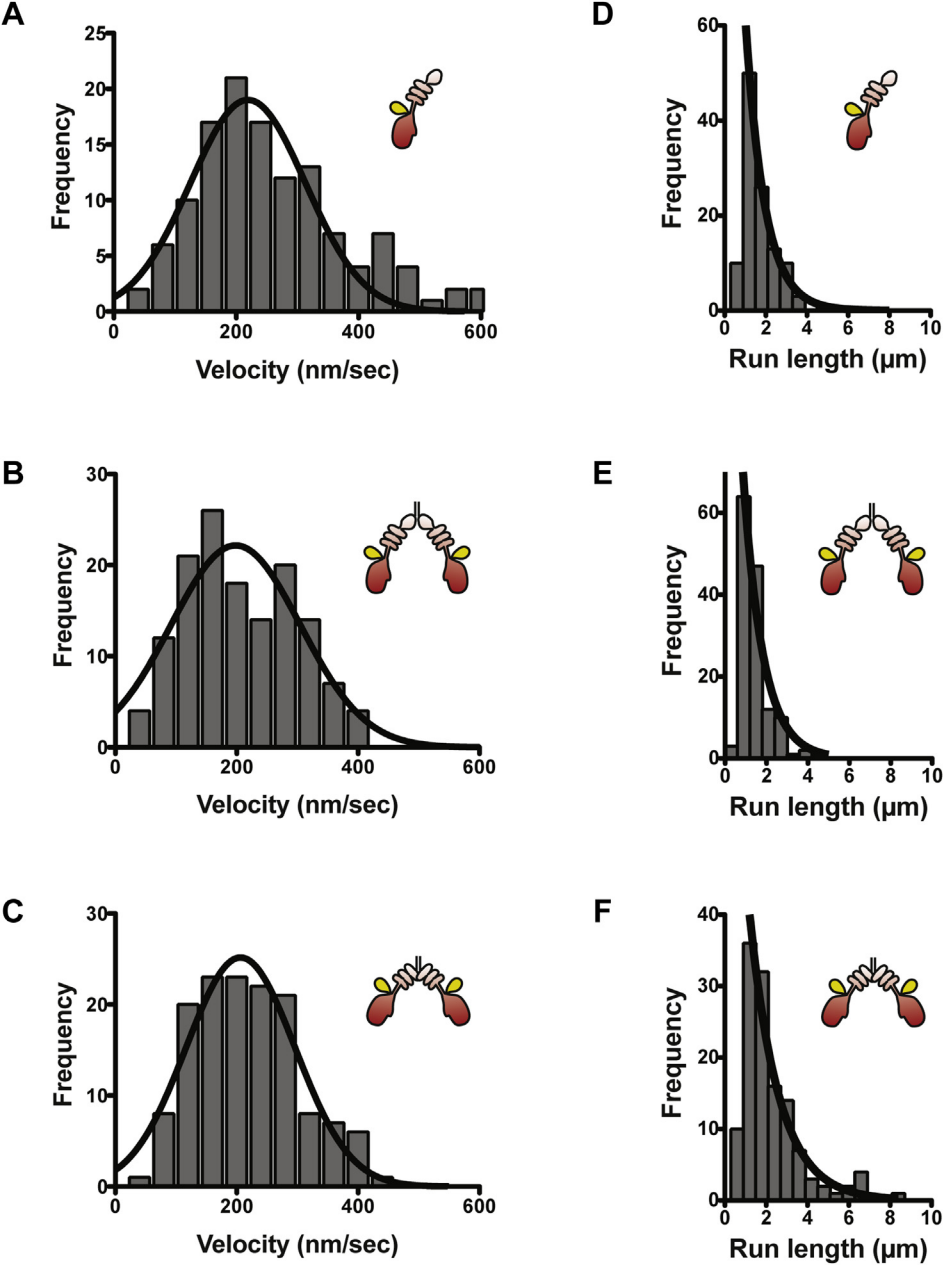
**Movement of HM19 constructs in living cells.** Cultured HeLa cells were transfected with Halo-HM19 constructs and stained with R110 Direct HaloTag ligands according to the “Experimental procedures” section. The movement in filopodia was then observed at 1 or 2 fps with a TIRF microscope by HILO illumination. The run length and velocities were determined as described in the “Experimental procedures” section. *A*–*C*, the histograms of velocities of Halo-HM19 constructs in filopodia. (*A*) R110-Halo-HM19^Full^WT, (*B*) R110-Halo-HM19^Full^LZ, and (*C*) R110-Halo-HM19^IQ3^LZ. The mean velocities were 0.263 ± 0.012 μm/s (SEM, n = 127) for R110-Halo-HM19^Full^WT, 0.209 ± 0.008 μm/s (SEM, n = 140) for R110-Halo-HM19^Full^LZ and 0.219 ± 0.008 μm/s (SEM, n = 128) for R110-Halo-HM19^IQ3^LZ. The distributions were fitted to a Gaussian equation (*solid lines*). The movement toward filopodial tip was analyzed. *D*–*F*, the histograms of run lengths of R110-Halo-HM19s in filopodia. (*D*) R110-Halo-HM19^Full^WT, (*E*) R110-Halo-HM19^Full^LZ, and (*F*) R110-Halo-HM19^IQ3^LZ. The *solid line* shows the best fit to a single exponential equation, *R*_*0*_e^−*r*/*λ*^. The calculated average run lengths for R110-Halo-HM19^Full^WT, R110-Halo-HM19^Full^LZ, and R110-Halo-HM19^IQ3^LZ were 0.91 ± 0.08 μm (SEM, n = 127), 0.95 ± 0.17 μm (SEM, n = 140), and 1.4 ± 0.2 μm (SEM, n = 128), respectively. The movement toward filopodial tip was analyzed. HILO, highly inclined and laminated optical sheet; HM19, human Myo19; LZ, leucine zipper; R110, rhodamine 110; TIRF, total internal reflection fluorescence.

**Figure 5:**
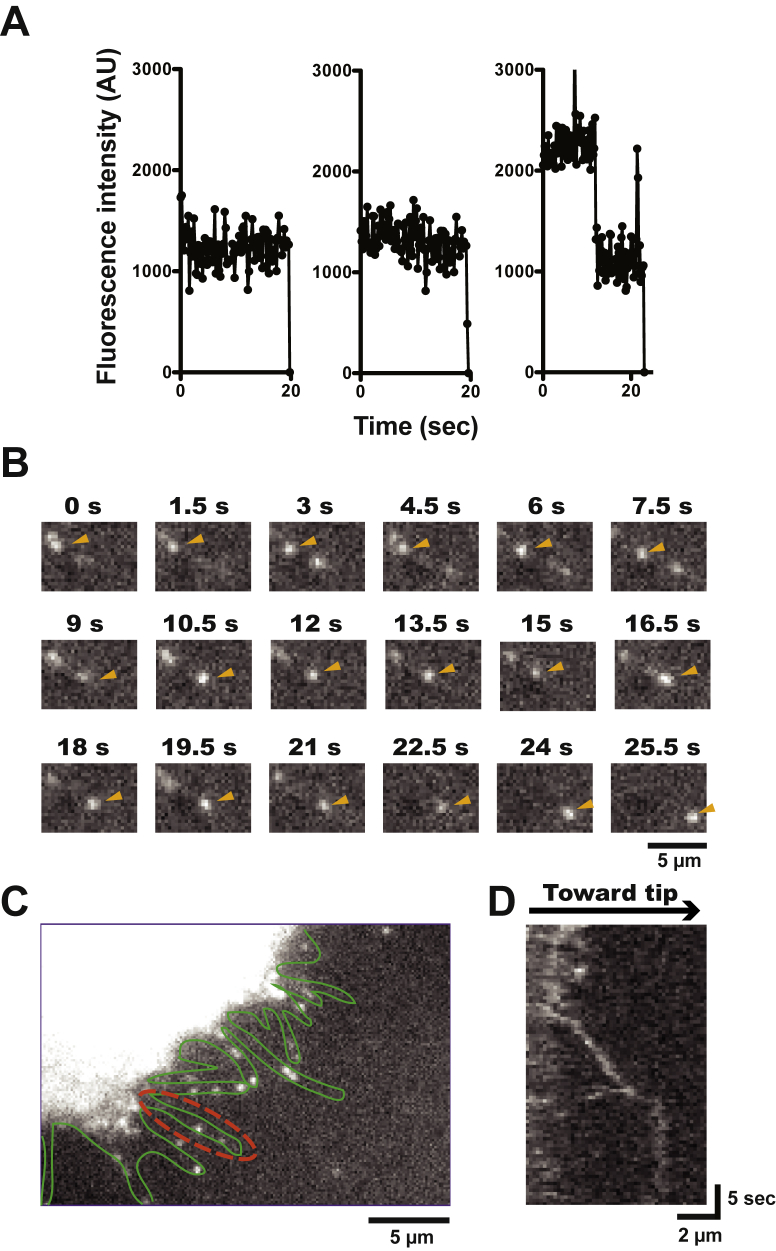
**Single-molecule imaging of HM19**^**Full**^**WT in living cells.** Cultured HeLa cells were transfected with Halo-HM19^Full^WT and stained with R110 Direct HaloTag ligands, and the movement of R110-Halo-HM19^Full^WT molecules in filopodia was observed with a TIRF microscope by HILO illumination. *A*, representative photobleaching of the fluorescent spots of R110-Halo-HM19^Full^WT. Most of the fluorescent spots show one-step photobleaching, but two-step photobleaching can also be found. The images were captured at 2 fps. *B*, time-lapse images of R110-Halo-HM19^Full^WT movement in living HeLa cells. Moving fluorescent spots are indicated by *arrowheads*. The images were captured at 2 fps. *C*, representative image of living HeLa cells. The profile of filopodia was shown in *green lines*. The *broken red line* shows a typical filopodia used for the kymograph analysis shown in (*D*). Kymograph shows the fluorescent spot continuously move in filopodia. HILO, highly inclined and laminated optical sheet; HM19, human Myo19; R110, rhodamine 110; TIRF, total internal reflection fluorescence.

**Figure 6:**
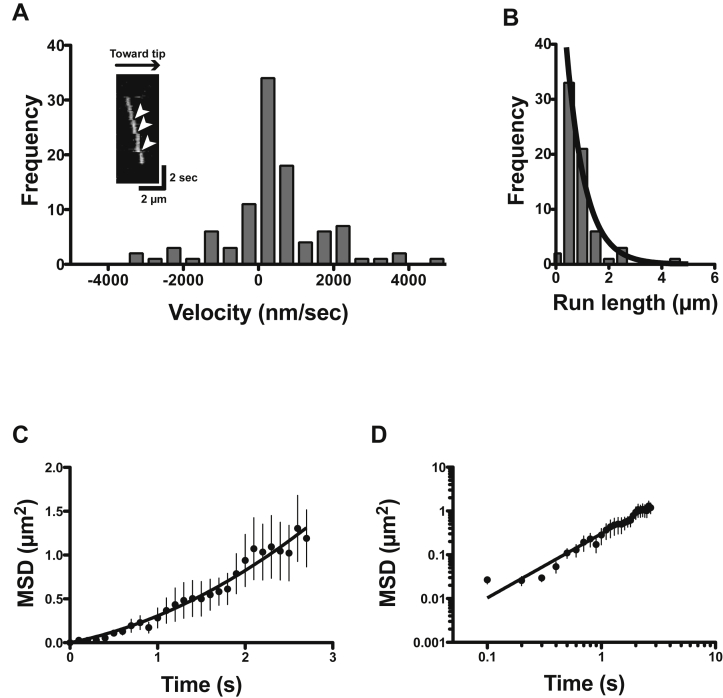
**Diffusive component and mean square displacement (MSD) analysis of the movement of HM19**^**Full**^**WT in living cells.***A*, velocity distribution of R110-Halo-HM19^Full^WT movement. R110-Halo-HM19^Full^WT movements in living HeLa cells were captured at fast frame rate (10 fps), and the velocities were determined from the kymographs (n = 106). The inset in (*A*) shows a typical kymograph of R110-Halo-HM19^Full^WT movement. By monitoring the movement with fast frame rate, occasional jumps were observed along with continuous directional movement (*arrowheads*). These jumps, presumably because of diffusion, were observed in both plus and minus directions. The forward and backward movements within filopodia are shown in the figure. It is expected that these fast diffusive components of plus and minus directions can be canceled out when the movement is monitored with slow frame rate. *B*, run length distribution of R110-Halo-HM19^Full^WT movement. *Solid line* shows the best fit to a single exponential equation, *R*_*0*_e^−r/*λ*^. The calculated average run length for R110-Halo-HM19^Full^WT was 0.70 ± 0.08 μm (SEM, n = 67). The movement toward filopodial tip was analyzed. *C*, MSD analysis for R110-Halo-HM19^Full^WT movement. The position of moving light spot was tracked using 2D fitting and tracking software, and the MSD was calculated. The plot is fitted with an equation of f(*t*) = 4*Dt* + *v*^*2*^*t*^*2*^ for 0 to 2.7 s (*solid line*, *v* = 0.33 ± 0.07 μm/s, *D* = 0.05 ± 0.02 μm^2^/s), where *v* = velocity; *t* = time; and *D* = diffusion coefficient. Error bars represent SEM (n = 9). *D*, log–log plot of MSD analysis for R110-Halo-HM19^Full^WT. The same data in (*C*) were plotted in the log–log axes and fitted with f(*t*) = *ct*^*α*^, where *c* = constant, *t* = time, and *α* = slope of the plot (*solid line*). The *α* value of the plot for R110-Halo-HM19^Full^WT is calculated to be 1.47 ± 0.08. Error bars represent SEM. HM19, human Myo19; R110, rhodamine 110.

Next, we asked whether HM19 can processively move in live cells at a single-molecule level. Halo-tagged HM19 constructs were expressed in HeLa cells, stained with rhodamine 110 (R110) and observed with TIRF microscope by HILO illumination (Figure 4, Figure 5, Figure 6 and S7–S11, and see also “Experimental procedures” section). The HILO illumination has great advantages that not only include being able to easily find the cell structures expressing the labeled protein but also being able to photobleach a wide range of cells for single-molecule observation. We studied the movement on filopodial actin tracks that are unidirectional with barbed ends at the tips of filopodia. We first examined the movement of forced dimer constructs, Halo-HM19^Full^LZ and Halo-HM19^IQ3^LZ in living cells showing notable accumulation of fluorescent signals at the filopodial tips at a single-molecule level, consistent with the confocal microscope observation of these constructs (Fig. S6).

We found many moving fluorescence spots in filopodia. Fig. S7 shows a typical two-step photobleaching of R110-Halo-HM19^Full^LZ and R110-Halo-HM19^IQ3^LZ. This suggests that the fluorescent spots of the LZ constructs contain two Halo moieties. We also measured the fluorescent intensity of each particles, and the distribution of intensities was analyzed (Fig. S8, *B* and *C*). There were two peaks in which the higher intensity was approximately double of the lower intensity, suggesting that the former contains two fluorophores and the latter contains one fluorophore, respectively. The result also suggests that R110-Halo-HM19^Full^LZ and R110-Halo-HM19^IQ3^LZ contain two Halo moieties. Fig. S9*A* shows representative successive snapshot images of the movement of R110-Halo-HM19^IQ3^LZ in filopodia. The kymograph analysis revealed the processive movement of R110-Halo-HM19^IQ3^LZ in filopodia (Fig. S9, *B* and *C*). The velocity and run length for R110-Halo-HM19^IQ3^LZ were 0.219 ± 0.008 μm/s and 1.4 ± 0.2 μm (mean ± SEM, n = 128), respectively (Fig. 4, *C* and *F* and Table 2). For R110-Halo-HM19^Full^LZ, the mean velocity and the run length were 0.209 ± 0.008 μm/s and 0.95 ± 0.17 μm (mean ± SEM, n = 140), respectively (Fig. 4, *B* and *E* and Table 2). The velocity of R110-Halo-HM19^Full^LZ in living cells was approximately three times higher than that of *in vitro* experiment of EGFP-HM19^Full^LZ using demembraned-cell system (Fig. 3). Considering the difference in the temperatures in two experiments (22 *versus* 37 °C), the difference between *in vitro* and *in vivo* velocities of HM19 is within an expected range of difference. The values of R110-Halo-HM19^Full^LZ were similar to those of R110-Halo-HM19^IQ3^LZ (Fig. 4, *C* and *F*), suggesting that the tail domain itself does not influence the velocity and run length of the movement of HM19. On the other hand, R110-Halo-HM19^Full^WT did not show apparent filopodial tip localization in contrast to R110-Halo-HM19^Full^LZ and R110-Halo-HM19^IQ3^LZ that showed notable localization at the filopodial tips (Figs. S3–S6). Figure 5*A* shows a typical photobleaching of R110-Halo-HM19^Full^WT. This suggests that the fluorescent spots of the HM19 WT construct contain predominantly one Halo moiety. The fluorescence intensity distribution of Halo-HM19^Full^WT showed that the monomeric form of Halo-HM19^Full^WT construct is dominant in contrast to those of LZ constructs (Fig. S8*A*). Quite interestingly, we were able to observe the movement of Halo-HM19^Full^WT on filopodia (Fig. 5*B*, and see also Movie S3) although this construct did not show notable localization at the tip of filopodia (Fig. 5*C*, and see also Fig. S5). Representative kymograph of the movement of Halo-HM19^Full^WT is shown in Figure 5*D*. In this kymograph, the molecule moves toward filopodial tips with occasional fast movement. The mean velocity and run length for R110-Halo-HM19^Full^WT were 0.263 ± 0.012 μm/s and 0.91 ± 0.08 μm (mean ± SEM, n = 127) (Fig. 4, *A* and *D* and Table 2), respectively. The velocity and run length were comparable to those of R110-Halo-HM19^Full^LZ, indicating that HM19^Full^WT can continuously move on actin track in filopodia without forced dimerization module. To further study the effect of the dimerization in the continuous movement of HM19, we next tested the tail-less HM19 construct without artificial dimerization motif (Halo-HM19^IQ3^). In contrast to the full-length construct, R110-Halo-HM19^IQ3^ failed to exhibit successive movement (Figs. S10 and S11) under a similar condition. This finding suggests that the tail domain facilitates the dimer formation of HM19, although our results do not directly support the dimerization at the tail domain. Supporting this notion, a certain population of the fluorescence spots of Halo-HM19^Full^WT showed two times higher fluorescent intensity of the monomeric form (Fig. S8*A*). It should be noted that a notable portion of the fluorescent spots of R110-Halo-HM19^Full^LZ and R110-Halo-HM19^IQ3^LZ showed approximately a half of the intensities of the dimer form. This suggests that some HaloTag ligands were not incorporated into Halo moieties in the experimental conditions.

**Table 2:** Run lengths and velocities of HM19 in living HeLa cells

HM19 constructs	Run length (*λ*)	Velocity
	μm	μm s^−^^1^
Halo-HM19^Full^WT	0.91 ± 0.08 (n = 127)	0.263 ± 0.012 (n = 127)
Halo-HM19^Full^LZ	0.95 ± 0.17 (n = 140)	0.209 ± 0.008 (n= 140)
Halo-HM19^IQ3^LZ	1.4 ± 0.2 (n = 128)	0.219 ± 0.008 (n = 128)
EGFP-HM19^Full^WT	0.85 ± 0.14 (n = 110)	0.216 ± 0.007 (n = 110)
EGFP-HM19^Full^LZ	0.94 ± 0.09 (n = 222)	0.185 ± 0.005 (n= 103)

Experiments were done at 37 °C (see the “Experimental procedures” section). Mean ± SEM was indicated.

To further analyze the movement of R110-Halo-HM19^Full^WT, we captured the images at a faster frame rate, 100 ms interval (10 fps). As shown in Figure 6*A*, R110-Halo-HM19^Full^WT shows a wide range of velocity distribution although the majority of velocities were 200 to 300 nm/s. The result suggests that R110-Halo-HM19^Full^WT movement includes the random motion on filopodial actin filaments (Movie S4). A similar distribution was also observed with the leucine-zippered tail-less-HM19 construct, Halo-HM19^IQ3^LZ (Fig. S10*B*). On the other hand, the tail and LZ-lacking HM19 construct, Halo-HM19^IQ3^, showed a wider velocity distribution without major velocity distribution at 200 to 300 nm/s range (Fig. S10*A*). This suggests that the movement of R110-Halo-HM19^IQ3^ is based upon random diffusion rather than active movement, whereas the movement of R110-Halo-HM19^Full^WT contains active movement that is similar to R110-Halo-HM19^IQ3^LZ with run length of ∼1 μm (Fig. 6*B*) in addition to random diffusion. These results suggest that the full-length HM19 construct in part forms a dimer without the LZ moiety in living cells. To address this notion, we tested another myosin class, myosin 10, which is known to make a dimer *via* the native coiled coil (Fig. S10, *C* and *D*). We found that myosin 10 without coiled coil, that is, monomeric myosin 10, also showed the movement similar to R110-Halo-HM19^IQ3^, whereas myosin 10 with native coiled coil showed predominantly directional movement with ∼1 μm/s, which is consistent with the velocity of myosin 10 in cells (40, 41). The results support our idea that the movement of R110-Halo-HM19^IQ3^ is random diffusion on actin filaments, whereas R110-Halo-HM19^Full^WT actively moves. The inset of Figure 6*A* shows a representative kymograph of the movement of R110-Halo-HM19^Full^WT. R110-Halo-HM19^Full^WT moved continuously toward the filopodial tips, and the movement demonstrated jumps at multiple points (represented by *arrowheads*), suggesting the presence of diffusive motion during the directional movement.

Figure 6*C* shows the mean square displacement (MSD) analysis of R110-Halo-HM19^Full^WT. The plot does not fit random motion: f(*t*) = 4Dt. When the plot was fitted with f(*t*) = 4*Dt* + *v*^*2*^*t*^*2*^, where *v* = velocity, *t* = time, and *D* = diffusion coefficient, the velocity and the diffusion coefficient were 0.33 ± 0.07 μm/s and 0.050 ± 0.024 μm^2^/s (SEM, n = 9), respectively. Although the apparent velocity of R110-Halo-HM19^Full^WT was similar to R110-Halo-HM19^IQ3^LZ (*v* = 0.31 μm/s, Fig. S11*B*), the diffusion coefficient of R110-Halo-HM19^Full^WT was three to four times larger than that of R110-Halo-HM19^IQ3^LZ (0.014 ± 0.010 μm^2^/s), which was approximately five times less than that of R110-Halo-HM19^IQ3^ (0.24 ± 0.01 μm^2^/s at 0.5–2.0 s, Fig. S11*A*). Figure 6*D* shows log–log plot of MSD over time. When the plot is fitted with f(*t*) = *ct*^*α*^, where *c* = constant, *t* = time, and *α* = slope, the *α* value for R110-Halo-HM19^Full^WT is calculated to be 1.47 ± 0.08 (SEM), which is between R110-Halo-HM19^IQ3^ (*α* = 1.17 ± 0.01, Fig. S11*C*) and R110-Halo-HM19^IQ3^-LZ (*α* = 1.74 ± 0.01, Fig. S11*D*). This suggests that the movement of R110-Halo-HM19^Full^WT is composed of slow directional movement with the velocity similar to the forced dimer construct and fast diffusion component. These analyses further support an idea that HM19^Full^WT without LZ can continuously move on actin in living cells (see Discussion section).

A potential experimental limitation of the use of HaloTag technology is that it is not fully assured whether HaloTag ligands bind all the expressed Halo-tagged protein molecules in cells. This implies that the moving single fluorescence spots may not always be single molecules of HM19s. To address this issue, we also examined the cellular movements of HM19 using EGFP-HM19^Full^WT and EGFP-HM19^Full^LZ in living HeLa cells (Figs. S12 and S13). While the number of filopodial tips having EGFP-HM19^Full^WT was fewer than those of EGFP-HM19^Full^LZ (Fig. S3), we found that EGFP-HM19^Full^WT also localized at the tip of the filopodia (Fig. S12). EGFP-HM19^Full^WT moved on filopodial actin tracks at a single-molecule level. The velocity and run length were 0.216 ± 0.007 μm/s (mean ± SEM, n = 110) and 0.85 ± 0.14 μm (mean ± SEM, n = 110) for EGFP-HM19^Full^WT and 0.185 ± 0.005 μm/s (mean ± SEM, n = 103) and 0.94 ± 0.09 μm (mean ± SEM, n = 222) for EGFP-HM19^Full^LZ, respectively (Fig. S13 and Table 3). These results support that the HM19^Full^WT single molecules can move processively on cellular actin tracks in living cells.

**Table 3:** Single-molecule parameters of EGFP-HM19^Full^LZ

ATP concentration	Step size	Dwell time (*τ*)	Run length (*λ*)	Velocity
	nm	s	μm	nm s^−^^1^
5 μM ATP^a^	ND^b^	ND	0.14 ± 0.06 (n = 34)	54.4 ± 4.8 (n = 34)
1 mM ATP^a^	ND	ND	0.18 ± 0.01 (n = 47)	95.1 ± 7.4 (n = 47)
2 μM ATP^c^	33.8 ± 1.3 (n = 91)	1.4 ± 0.1 (n = 46)	0.17 ± 0.01 (n = 41)	ND

Experiments were done on single actin filaments in the presence of the indicated ATP concentration (see the “Experimental procedures” section). Mean ± SEM was shown.

a EGFP fluorescence was monitored.

c Qdot was used.

b ND, not determined.

### Single-molecule movement and stepping of HM19 *in vitro*

**Figure 7:**
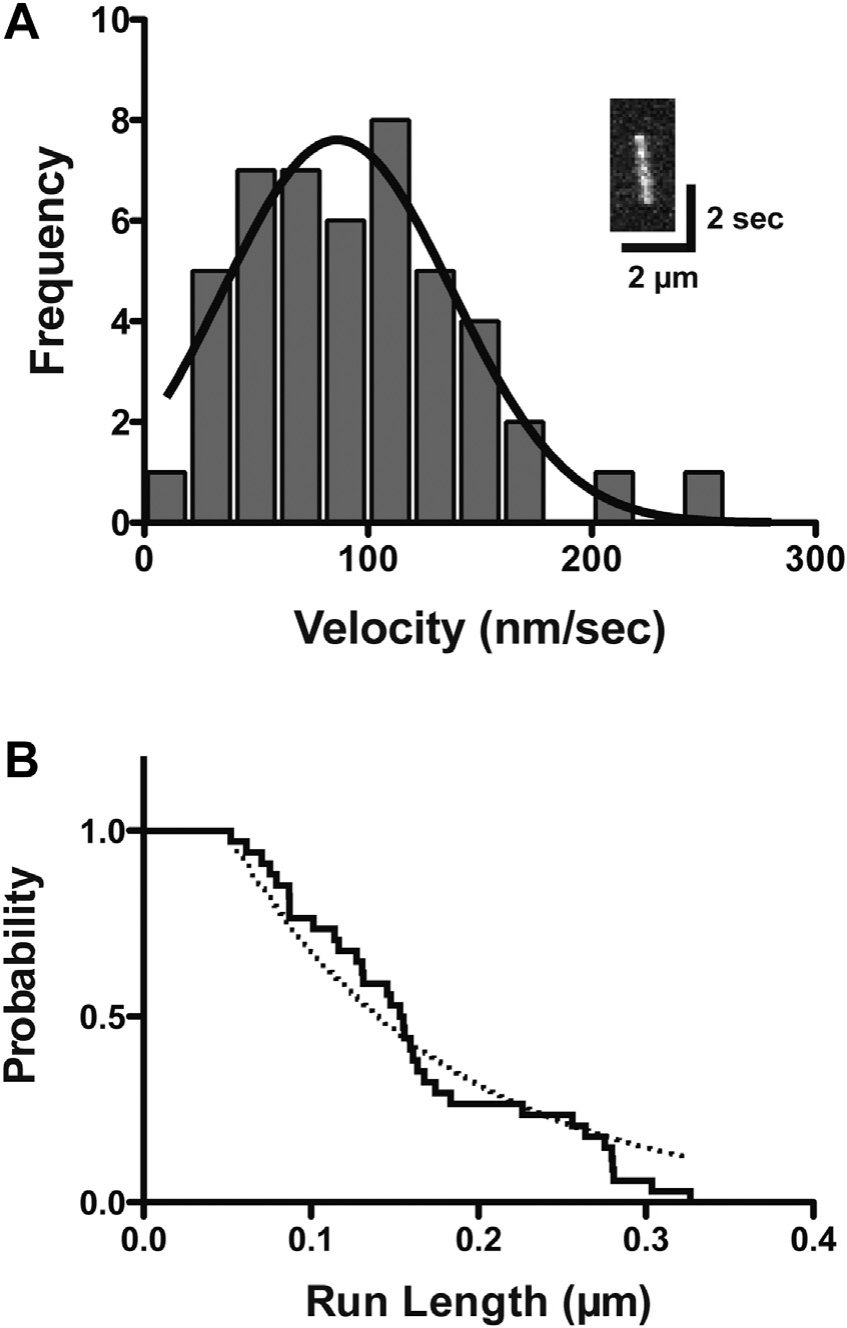
**Velocity and run length of HM19**^**Full**^**LZ on single actin filaments *in vitro*.** The single-molecule movement of purified EGFP-HM19^Full^LZ on single actin filaments was observed with a TIRF microscope. Experiments were carried out in the presence of 1 mM ATP using glass flow chambers in which actin filaments were immobilized (see the “Experimental procedures” section), and the fluorescence images were captured at 10 fps. *A*, the velocity of EGFP-HM19^Full^LZ on single actin filaments. The mean velocity was 95.1 ± 7.4 nm/s (SEM, n = 47). The *solid line* shows the best fit to a Gaussian equation. *Inset* shows a typical kymograph of the EGFP-HM19^Full^LZ movement. *B*, run length of EGFP-HM19^Full^LZ on single actin filaments. The graph was shown with a Kaplan–Meier survival curve. *Broken line* shows the best fit to the single exponential equation as described for Figure 3. The average run length was 0.18 ± 0.01 μm (SEM, n = 47). EGFP, enhanced GFP; HM19, human Myo19; LZ, leucine zipper; TIRF, total internal reflection fluorescence.

While aforementioned results indicate the continuous movement of HM19 at single-molecule level in cells, the best approach to obtain conclusive evidence of processive movement of HM19 is *in vitro* single-molecule motility analysis (Fig. 7). To observe the HM19 movement, we made flow chambers in which single actin filaments were immobilized on the glass surface and monitored the movement of isolated EGFP-HM19^Full^LZ molecules in physiological ATP concentration. The mean velocity in the presence of 1 mM ATP was 95.1 ± 7.4 nm/s (mean ± SEM, n = 47) (Fig. 7*A*), which was 1.7 times faster than the velocity at 5 μM ATP in a similar condition (Table 3). The average run length at 1 mM ATP was 0.18 ± 0.01 μm (n = 47) (Fig. 7*B*).

**Figure 8:**
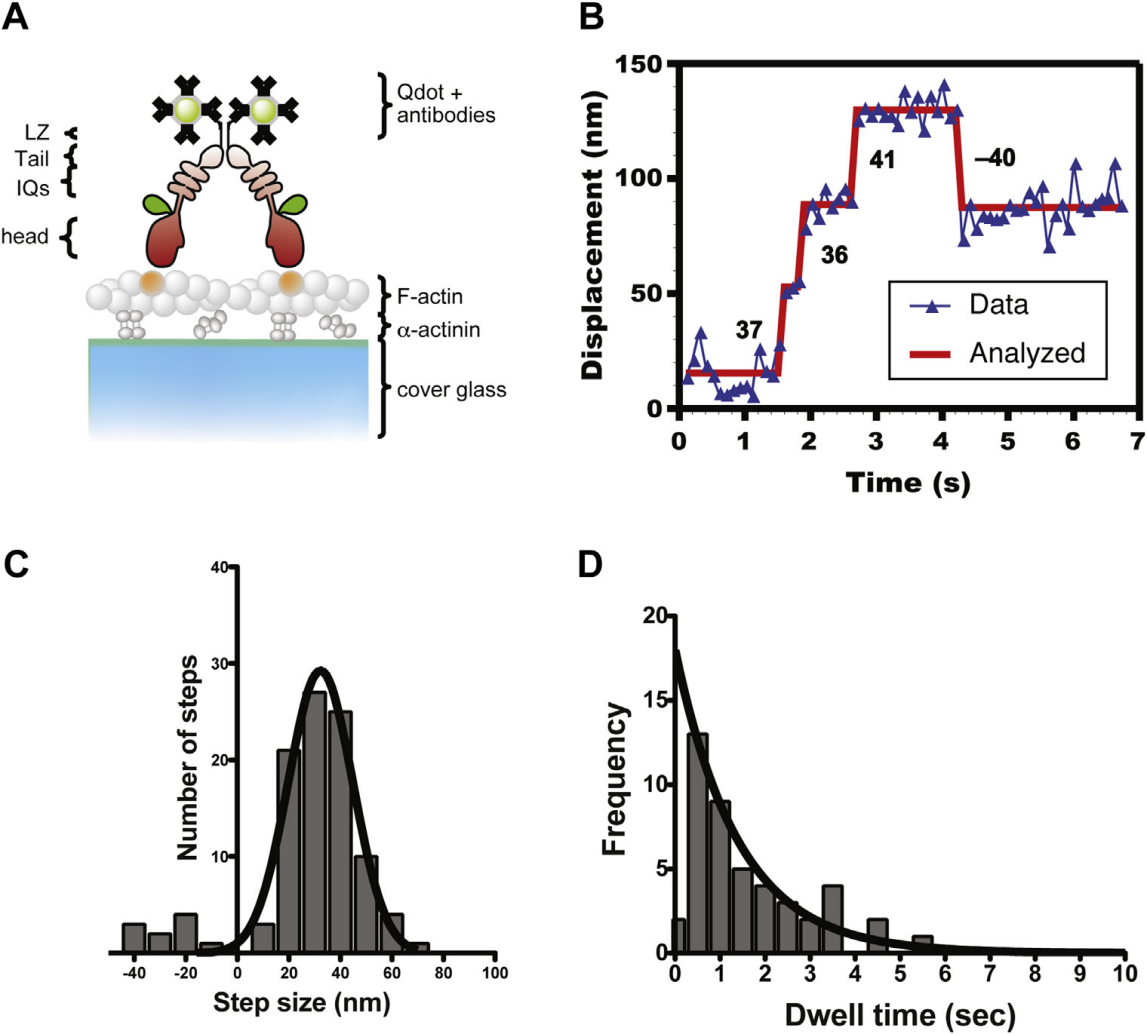
**Step-size and dwell time distribution of HM19**^**Full**^**LZ-Qdot on single actin filaments.** The movement of EGFP-HM19^Full^LZ-Qdot525 (see the “Experimental procedures” section) on single actin filaments was observed with a TIRF microscope. *A*, schematic drawing of EGFP-HM19^Full^LZ-Qdot525. The Qdots were attached to the C-terminal c-Myc tag of EGFP-HM19^Full^LZ through first (anti-c-Myc) and second (antimouse Fab’) antibodies. *B*, a representative trace of EGFP-HM19^Full^LZ-Qdot525 stepping. The experiment was carried out in the presence of 2 μM ATP, and the fluorescence images were captured at 10 fps. *Solid line* shows the best fit to the trajectory. The number in the panel is the displacement of each step in nanometer. *C*, step size distribution of EGFP-HM19^Full^LZ-Qdot525. The mean step size of forward and backward steps is 33.8 ± 1.3 nm (mean ± SEM, n = 91) and −27.2 ± 3.3 nm (SEM, n = 10), respectively. *Black solid line* shows the best fit to a Gaussian equation. *D*, dwell time distribution of EGFP-HM19^Full^LZ-Qdot525 in the presence of 2 μM ATP. *Solid line* shows the best fit to a single exponential equation, *ke*^−*kt*^, where *t* is time, and *k* is rate constant (the first bin is excluded from the fitting). The average waiting time (*τ* = 1/*k*) is 1.4 ± 0.1 s (SEM, n = 46). EGFP, enhanced GFP; HM19, human Myo19; LZ, leucine zipper; Qdot, quantum dot; TIRF, total internal reflection fluorescence.

To determine the HM19^Full^LZ stepping, Qdot was attached to the C-terminal end of an isolated HM19^Full^LZ through anti-c-Myc antibodies (Fig. 8*A*). We observed the successive HM19^Full^LZ-Qdot movement on single actin filaments in the presence of 2 μM ATP. By tracking the center position of HM19^Full^LZ-Qdots using FIONA technique (9), we determined the step sizes of HM19^Full^LZ (Fig. 8*B* and Movie S5). Figure 8*C* shows the step size distribution of HM19^Full^LZ-Qdot. The distribution was symmetrical, and the mean step size of HM19^Full^LZ-Qdot was 33.8 ± 1.3 nm (mean ± SEM, N_steps/Qdots_ = 91/26) for forward step and –24 ± 3.3 nm (SEM, N_steps/Qdots_ = 10/6) for backward step. The mean forward step was slightly shorter than a half pitch of F-actin helix (∼36 nm). In most cases, the back step was only single steps, and we did not observe successive back steps. Figure 8*D* is dwell time distribution of HM19^Full^LZ-Qdot at 2 μM ATP. The average waiting time was 1.4 ± 0.1 s (SEM, n = 46). The average waiting time gives the single turnover rate of 0.7 s^−1^ at 2 μM ATP. Run length distribution of HM19^Full^LZ-Qdot gave the average run length of 0.17 ± 0.01 μm (SEM) at 2 μM ATP, which was similar to the run length of EGFP-HM19^Full^LZ at 1 mM ATP (Fig. 7*B* and Table 3). These results indicate that HM19^Full^LZ-Qdot moves four to five steps on average at the physiological concentration of ATP on single actin filaments and takes 2.8 steps per second, that is, ∼2.8 s^−1^ at 22 °C. This value is consistent with the *V*_max_ of the actin-activated ATPase activity for monomeric truncated HM19 (23, 24).

## Discussion

Although it is well known that kinesin–dynein motor proteins are associated with mitochondrial movement (39), several myosins such as nonmuscle myosin 2, myosin 5a, myosin 6, and Myo19 (19), are found adjacent to mitochondria (42). However, the role of this myosin is still unknown. Since Myo19 is highly colocalized with mitochondria, it has been thought that it plays a role in mitochondrial dynamics and movement (11). Myo19 can be found at the filopodial tips (13, 15). However, it is unclear whether Myo19 can actively and processively move to the filopodial tips or if it localizes at the filopodial tips by other means such as diffusion or movement driven by other motor proteins. Our objective of the present study is to clarify whether HM19 is a processive motor that can actively transport mitochondria. In the present study, we showed for the first time that HM19 can processively move within filopodia to the tips and transport mitochondria through a processive manner. This processive movement of HM19 agrees with the high duty ratio of tail-truncated HM19 by kinetic analyses (23, 24), as high duty ratio is one of the critical features to account for processive movement of motor proteins (1, 43).

We found that the isolated mitochondria membrane fraction is localized at actin tracks on demembraned cells (Fig. S2). This suggests that endogenous HM19 on mitochondria membranes attaches to actin filaments. This view is supported by the result that the mitochondria membranes were prepared from the cells expressing Halo-HM19^Full^LZ; the forced dimer of HM19 not only localized at the actin tracks but also moved on the cellular actin tracks in the presence of ATP (Fig. 3). However, the mitochondrial membrane fraction with endogenous HM19 did not move on the cellular actin tracks in the presence of ATP in *in vitro* system. It is assumed that endogenous HM19 is present on mitochondria from untransfected cells (13). Therefore, a question is why the endogenous HM19 did not apparently support the isolated mitochondrial movement. It is plausible that the majority of endogenous HM19 molecules on isolated mitochondrial membrane *in vitro* are “inhibited,” presumably because of its monomeric state.

It has been known that various myosin superfamily members are regulated through its binding molecules such as melanophilin for myosin 5a (44, 45), Dab2, phosphatidylinositol 4,5-bisphosphate for myosin 6 (30), and phosphatidylinositol 3,4,5-trisphosphate for myosin 10 (4). Therefore, it is plausible that HM19 binding partners regulate the motor activity of the endogenous HM19 including dimer formation.

To directly evaluate the transporter activity of HM19, we studied the movement of EGFP-HM19-forced dimer at single-molecule level and found that it processively moves on cellular actin tracks in demembraned cells (Fig. 3). This implies that HM19-forced dimer can be a processive motor suitable for mitochondria transport. The velocity of EGFP-HM19-forced dimer on demembraned cells showed two velocity peaks, which were similar to those of mitochondria membranes obtained from the cells expressing HM19-forced dimer. This result suggests that HM19-forced dimer supports the movement of isolated mitochondrial membrane. We think that the slower velocity was also driven by HM19 because the mitochondria membranes obtained from nontransfected cells did not show the movement. The velocity of ∼60 nm/s of EGFP-HM19-forced dimer is similar to the velocity (40–50 nm/s) of multimolecule *in vitro* motility of mouse Myo19 IQ3 (tail-truncated mouse Myo19) described by Lu *et al.* (23). Halo-HM19-forced dimers localize at filopodial tips in live cells, whereas Halo-HM19^Full^WT did not show notable localization at the filopodial tips (Fig. S5). On the other hand, the tail-truncated Halo-HM19-forced dimer localizes at filopodial tips, but not with mitochondria, which is consistent with the previous reports that the tail domain of HM19 is responsible for mitochondrial binding (11, 13, 14). A critical question is whether HM19 actually moves toward filopodial tips by its own motor activity. In order to address this question, we studied intrafilopodial movement of HM19 at a single-molecule level in living cells by using TIRF microscopy. Halo-HM19-forced dimers continuously moved within filopodia in living cells. Kymograph analysis revealed that the movement is unidirectional, that is, toward filopodial tips. Since the tip of filopodia is plus end of actin bundles (46), the result is consistent with the previous finding that showed HM19 is a plus-directed myosin (23). Halo-ligand provides more intense fluorescence, which is advantageous for tracking the movement of HM19, whereas a single fluorophore containing moving spots may contain HM19 molecules without bound Halo ligand, thus possibility of having multiple HM19 molecules. To circumvent this problem, we employed EGFP-HM19^Full^LZ (Fig. 3). Single fluorescent EGFP spot of EGFP-HM19^Full^LZ processively moved on actin track. The result further supports processive movement of a single-molecule HM19 dimer.

Since HM19 does not have a coiled-coil domain (2, 10), it is thought that HM19 is a monomeric myosin. However, it is known that the two heads of myosin can concertedly interact with actin to produce processive movement upon actin through a hand-over-hand mechanism (9). Quite interestingly, we observed a number of fluorescent spots of Halo-HM19^Full^WT molecules moving in filopodia (Figs. 4 and 5), although Halo-HM19^Full^WT did not show apparent localization at filopodial tips under confocal microscope (Fig. S5). For Halo-HM19-forced dimers, the fluorescence intensity at filopodial tips was 6 to 10 times brighter compared with Halo-HM19^Full^WT (Fig. S6). This indicates that Halo-HM19-forced dimers can more efficiently move to filopodial tips. Note that the velocity of HM19-forced dimer toward the tip of filopodia in living cells (∼200 nm/s) is much faster than that of isolated EGFP-HM19-forced dimer (∼60 nm/s) *in vitro*. The difference in the velocity is consistent with the temperature difference of ATPase activity between *in vitro* and *in vivo* conditions (Q10 of HM19 = ∼2.5 calculated from temperature difference [22 *versus* 37 °C]; data not shown). On the other hand, the kymograph analysis for Halo-HM19^Full^WT shows processive movement in filopodial actin tracks in living cells (Figs. 4 and 5), and the velocity of Halo-HM19^Full^WT was essentially the same as that of Halo-HM19-forced dimers (Fig. 4). This indicates that Halo-HM19^Full^WT moves with its own motor activity. The run lengths of both Halo-HM19-forced dimers and Halo-HM19^Full^WT in living cells were ∼2.5 times longer than that of isolated EGFP-HM19-forced dimer *in vitro* (∼1 *versus* ∼0.4 μm). This is likely because of the difference in cells and *in vitro* environments, such as higher effective concentration of Myo19 in cells. A critical question is why HM19^Full^WT is not accumulated at the filopodial tips despite the fact that we found processive movement of HM19^Full^WT in living cells. Taking into account these findings, we propose the following hypotheses. HM19^Full^WT is predominantly inactive within cells, presumably in its monomeric form. However, a certain population of HM19^Full^WT is activated, presumably because of the conversion from monomer to dimer by unknown mechanism. Supporting this view, it was found that a part of fluorescent spots of Halo-HM19^Full^WT showed two-step photobleaching, suggesting the presence of two fluorophores, although the fluorescent spots of Halo-HM19^Full^WT contain mostly one fluorophore (Figs. 5*A* and S8). Moreover, we found that the run length of HM19^Full^WT is similar to HM19^Full^LZ, suggesting that the activated form of HM19^Full^WT, which is presumably its dimer form, moves similar to the forced dimer of HM19. While these findings suggest that HM19^Full^WT can form a dimer in cells, it is likely that the produced dimer is not highly stable in the same way as forced-dimer constructs of HM19. Supporting this view, we found that the accumulation of HM19^Full^WT at filopodial tips was much lower than that of HM19-forced dimer (Figs. S6 and S12). We think that the HM19^Full^WT can form a dimer while it moves on actin filaments as shown previously for the tail-truncated myosin 10 that the dimer formation can be facilitated *via* myosin head–actin interaction (33, 47). However, once it dissociates from actin track, the dimer of the HM19^Full^WT is disassembled into monomers and diffused away from the actin track. On the other hand, the stable forced dimer of HM19^Full^, that is, HM19^Full^LZ, can rebind to the actin track and move further toward filopodial tips, thus accumulating at the tips. In addition, since the population of HM19^Full^WT dimer is likely to be much less than the HM19-forced dimer, it is anticipated that the number of HM19^Full^WT molecules processively moving on actin track is much fewer than that of forced dimer constructs. These can explain much lower accumulation of HM19^Full^WT at the filopodial tips.

Analysis of MSD data further supports this view (Figs. 6 and S11). In MSD analysis with log–log axes, the slope (*α*) = 1 indicates that the movement is purely by diffusion and the (*α*) = 2 indicates the movement with a constant velocity without diffusion. In the case of myosin 5a HMM movement on single actin filaments *in vitro*, the value is estimated as *α* = 1.9 (48). This indicates that myosin 5a moves at a constant velocity without diffusion *in vitro*. On the other hand, the *α* values were 1.2, 1.7, and 1.5, for HM19^IQ3^, HM19^IQ3^LZ, and HM19^Full^WT, respectively (Figs. 6*D* and S11). This result suggests that the movement of HM19^IQ3^ is dominantly diffuse. The movement of HM19^Full^WT is more directional than that of HM19^IQ3^, but more diffusive than HM19^IQ3^LZ, which shows constant directional movement. This is in good agreement with the result of the calculated velocity = ∼330 nm/s of HM19^Full^WT from the MSD analysis, which contains significant nondirectional diffusive movement. Interestingly, time *versus* MSD of HM19^IQ3^ showed a nonlinear relationship, especially in a short period (Fig. S11*A*, *inset*). This suggests that HM19^IQ3^ single molecules can exhibit directional movement in certain short periods although HM19^IQ3^ moves predominantly with a simple diffusion. This may indicate that the tail-truncated single-headed HM19 has the ability to move on filopodial actin filaments, similar to the single-headed myosin 9 (49). It was reported previously that KIF1A, one of the kinesin family members, shows simple diffusive movement with some biased diffusion (50). Therefore, the movement of HM19^IQ3^ may also contain a biased diffusion component.

EGFP-HM19^IQ3^ shows virtually no filopodial tip accumulation (data not shown), whereas HM19^Full^WT showed some filopodial tip accumulation in live cells (Fig. S12). This is consistent with the MSD analysis described above (Fig. 6). The result also suggests that the tail domain contributes to a dimer formation. There is no predictable coiled-coil domain in the tail domain of HM19, and a question is how HM19 can form a dimer in cells. One possibility is that HM19 may form a dimer through the binding of its partner molecules. The reason for this possibility is that one of the potential binding proteins is Miro1/2, which is a protein that is known to recruit and stabilize HM19 with mitochondria. However, the direct binding of HM19 to Miro1/2 protein remains to be fully clarified (16). Alternatively, C-terminal tail domain of HM19 may directly dimerize through the binding of some small molecules as it is reported that phosphatidylinositol 3,4,5-trisphosphate binding at the tail of myosin 10 facilitates dimer formation (29) and phosphatidylinositol 4,5-bisphosphate binding at the C-terminal tail region of myosin 6 promotes dimer formation (30). Further studies are required to clarify the regulation mechanism of dimer formation of HM19.

In order to further clarify the characteristics of processive movement of HM19 at the molecular level, the movement and stepping of HM19 was studied with *in vitro* single-molecule motility assay. Our result clearly indicates that HM19 dimer processively moves on single actin filaments (Fig. 7). To further analyze the processive movement of HM19, we studied the successive stepping of HM19 using Qdot-attached HM19 dimer (Fig. 8*A*). The HM19^Full^LZ single molecule moved on single actin filaments for multiple successive steps (Fig. 8*B*). These results are consistent with the results obtained with EGFP-HM19^Full^LZ in cells and demembraned cells and support that an HM19 dimer can processively move on actin filaments. The average step size of HM19^Full^LZ was ∼34 nm. It has been thought that the length of the IQ domain of myosin is critical for the lever-arm length, hence its step size (51). HM19 has three IQ motifs that are calculated to be 11 to 12 nm of possible lever arm (52). In the case of myosin 10 having three IQ motifs, an SAH domain after the IQ domain serves as an extension of lever arm to explain its step size of 30 to 36 nm (33). There is no SAH sequence after the IQ domain of HM19, and it is obscure how HM19 shows large steps. It might be possible that the tail domain in addition to the IQ domain contributes to the step size of HM19^Full^LZ construct since the forced dimerization module is located at the C-terminal end of the molecule. However, since HM19^IQ3^LZ can also processively move toward filopodial tips, it is less likely that the tail domain is required for processive movement of HM19 in cells. Further study into the long lever arm of HM19 is required.

The run length of HM19^IQ3^LZ on single actin filaments *in vitro* was ∼180 nm (Fig. 7*B*). This is significantly shorter comparing with other processive myosins such as myosin 5a and myosin 10 (47, 53). A possible explanation is weak gating effect of HM19. Based upon the run length of HM19^IQ3^LZ, the duty ratio of HM19^IQ3^LZ is estimated to be ∼0.6 with the method previously used for myosin 5a (54). This finding suggests that single-molecule HM19 is not a great motor to transport cargos, such as mitochondria over a long distance. This conclusion is consistent with the studies by Usaj and Henn (24) and Shneyer *et al.* (15). Conversely, since it is highly likely that a large cargo such as mitochondria associates with a number of HM19 molecules, the assumption is that HM19 molecules can concertedly support successive movement of mitochondria in cells. If a single molecule of HM19 can move mitochondria for a long distance, many molecules associated with a single mitochondrion may interfere with each other for the movement. Our results indicate that HM19^Full^LZ takes the step size of ∼34 nm and moves on single actin filaments at ∼95 nm/s at 1 mM ATP. This indicates the single stepping rate of 2.8 s^−1^ at 22 °C. This rate is the same as 2.8 s^−1^ of the reported ATPase rate with HM19^IQ3^, a monomeric form at 25 °C (24). This further supports the idea that HM19 has a weak gating effect, in which the two heads of HM19 dimer may not influence each other to decrease the cycling rate.

In conclusion, we observed single-molecule movement of HM19 for the first time in *in vitro* and in cells and found that HM19 dimer moved processively on actin structure *in vitro* and in cells. We also demonstrated that the WT of HM19 without a forced dimerization module is capable of moving on filopodial actin tracks, presumably because of dimerization of HM19 in cells. Finally, we demonstrated that the C-terminal tail region of HM19 is critical for such movement of HM19 in cells.

## Experimental procedures

HM19 complementary DNA (cDNA) was purchased from GeneCopoeia (NM_001163735.1). MitoTracker, Alexa Fluor phalloidins, and Qdot (Q11041MP) were purchased from Invitrogen. HaloTag cloning starter system and HaloTag ligands were purchased from Promega. Actin was purified from rabbit skeletal muscle according to Spudich and Watt (55). Recombinant CaM (CALM2) (56, 57) was prepared from *Escherichia coli* as described previously (58).

Recombinant full-length HM19 (HM19^Full^WT) (including 1–970 of Myo19 amino acids) was subcloned into pEGFP-C1 (Clontech) to make EGFP-HM19^Full^WT. The stop codon of EGFP-HM19^Full^WT was removed by PCR site-directed mutagenesis (59) using Pfu Ultra II (Agilent Technologies), and an oligo DNA containing two sets of GCN4 LZ sequence (60) was then inserted to make EGFP-HM19^Full^LZ. The sequence after the end of HM19^Full^WT was SGTTHSYTSMKQLEDKVEELLSKNYHLENEVARLKKLVGERTSMKQLEDKVEELLSKNYHLENEVARLKKLVGERTSGSEL. For *in vitro* single-molecule motility assay of HM19, the stop codon of EGFP-HM19^Full^LZ was removed, and c-Myc/FLAG sequences (amino acid: EQKLISEEDL/DYKDDDDK) were introduced at the 3′ end. The N-terminal EGFP sequence was converted to HaloTag sequence (61) to make Halo-HM19^Full^WT and Halo-HM19^Full^LZ. The Halo-HM19^IQ3^ and Halo-HM19^IQ3^LZ constructs (including 1–834 of HM19 amino acids) were made by removing 835 to 970 amino acids from Halo-HM19^Full^WT and Halo-HM19^Full^LZ, respectively. The sequence of HM19^IQ3^LZ after the end of 834th amino acid was GTTHSYTSMKQLEDKVEELLSKNYHLENEVARLKKLVGERTSMKQLEDKVEELLSKNYHLENEVARLKKLVGERTSGSEL.

### Preparation of mitochondria membranes

HMF was prepared according to Shneyer *et al.* (13) with slight modifications. Briefly, ∼1 × 10^7^ (∼50 μl of the cell volume) of untransfected or Halo-HM19-transfected HEK293T cells were homogenized by pipetting in a homogenizing buffer (120 mM KCl, 20 mM Hepes–KOH [pH 7.4], 1 mM EDTA, 0.25 M sucrose, and protease inhibitor cocktail [catalog no.: 25955-24; Nakarai Tesque] containing 100 μM 4-(2-aminoethyl)benzenesulfonyl fluoride hydrochloride, 80 nM aprotinin, 1.5 μM E-64, 2 μM leupeptin, 5 μM bestatin, and 1 μM pepstatin A), followed by gentle sonication for 10 s. The cells were centrifuged at 1000*g* (TOMY AR015-24 rotor) for 1 min to pellet the cell debris. The supernatant was centrifuged again at 10,000*g* for 20 min to precipitate HMF, and the HMF pellet was resuspended in 100 μl of homogenizing buffer. For double staining of R110-Halo-HM19 with mitochondria vesicles, 10 μM MitoTracker Red FM (Thermo Fisher Scientific) was mixed with the HMF prepared from R110-Halo-HM19^Full^LZ transfected HEK293T cells. The HM19^Full^LZ-HMF was washed thrice with a homogenizing buffer by centrifugation at 10,000*g* for 20 min to remove unbound MitoTracker and resuspended in the same buffer. The aliquots of the HMF were flash-frozen by liquid nitrogen and stored at −80 °C.

### Preparation of purified HM19 protein

A nonmuscle-type myosin light chain (MRLC12A) cDNA was obtained from a human kidney cDNA library and transferred to pEB Multi vector (Fujifilm Wako Chemicals). HEK293T cells (Invitrogen) were transfected with the vector DNA, and neomycin-resistant MRLC12A-expressing HEK293T cells were created. EGFP-HM19^Full^LZ was then transiently expressed in MRLC12A-expressing HEK293T cells by a transfection of recombinant Myo19 heavy chain cDNAs using PEI Max (Polysciences, Inc) and then purified using anti-FLAG antibody-agarose resins (SIGMA). In short, 6 × 10^7^ cells were harvested at 24 h after transfection and homogenized in a lysis buffer (0.25 M KCl, 50 mM Hepes–KOH [pH 7.5], 5 mM EGTA, 15 mM β-mercaptoethanol, 1 mM ATP, 2% CHAPS, and protease inhibitor cocktail [described previously]). The suspension was mixed with anti-FLAG antibody-agarose resins, and the tube containing the suspension was rotated at 4 °C for 1 h. After two washes with a wash buffer (0.12 M KCl, 12.5% trehalose, 1 mM EGTA, 15 mM β-mercaptoethanol, 2% CHAPS, and protease inhibitor cocktail), the protein was eluted with an elution buffer (the wash buffer containing 0.1 mg/ml FLAG peptide). The eluted fractions were flash-frozen by liquid nitrogen and stored at −80 °C.

### Labeling of Halo-HM19s with HaloTag ligands and mitochondria staining in living cells

HeLa or HEK293T cells were cultured with Dulbecco’s modified Eagle's medium in 3.5 cm glass bottom dish (Matsunami Glass; D35-14-1-U), and cells were transfected with Halo-Myo19s using Lipofectamine 3000 (Invitrogen) according to the manufacturer’s protocol. After 16 to 24 hours, the medium was removed and the dish was washed with Leibovitz-15 medium (Gibco) plus 10% fetal bovine serum, followed by the addition of 0.1 μM R110 Direct HaloTag ligands at 37 °C for 15 min. Following thrice washes with Leibovitz-15 medium, the dish was incubated at 37 °C for 30 min in Leibovitz-15 medium. After thrice washes with FluoroBrite (Gibco), the cells were incubated at 37 °C in a CO_2_ incubator before observation. For mitochondria staining, the cells were incubated in a medium containing 0.2 μM MitoTracker for 30 min at 37 °C in a CO_2_ incubator.

### Qdot labeling of HM19 and fluorescent labeling of single actin filaments

For the step size and dwell time determinations, Qdot 525 conjugated with goat F(ab’)_2_ antimouse immunoglobulin G (H + L) were mixed with anti-c-Myc monoclonal antibody (Clontech) as described previously (33, 34). F-actin was labeled by Alexa Fluor 568 Phalloidin at a 1:1 ratio overnight and stored at 4 °C. For single-molecule experiments with Qdot-labeled HM19, 7.5 μM labeled and unlabeled actins were mixed at the ratio of 1:10, and the mixture was diluted to 1 μM just before the experiments.

### Demembraned cell preparation

U2OS cells were treated with an extraction buffer containing 30 mM imidazole, pH 7.5, 70 mM KCl, 1 mM EGTA, 2 mM MgCl_2_, 0.5% Triton X-100, 4% polyethylene glycol (mol weight = 8000), and 50 nM Alexa Fluor 568 phalloidin (Invitrogen) for 30 min on ice as described previously (33). Following three washes with PBS, all the samples were incubated on ice before observation of single-molecule assays.

### Single-molecule motility assay with TIRF microscope

The movement of R110-Halo-HM19s in living cells was observed with a hand-built TIRF/HILO setup (62) using an inverted Nikon Ti2 Eclipse microscope equipped with an objective lens (100× APO TIRF/numerical aperture [NA] = 1.49), a 488 nm Argon-ion laser system (Melles Griot), a back-illuminated electron multiplier charge-coupled device camera (iXON DU-897; Andor Technology), and temperature control system (model INU-KRi; TOKAIHIT). The fluorescent light of cultured cells expressing labeled HM19 proteins was monitored using Nikon NIS Elements software, and the movements of HM19 were recorded after proper photobleaching. The experiments were conducted at 37 °C. The movement of isolated EGFP-HM19^Full^LZ on demembraned cells and single actin filaments was observed with a hand-built TIRF setup using an Olympus IX71 microscope, an objective lens (PlanApo-N ×60, NA = 1.49, oil), a 488 nm argon laser (FITEL HPU50101; Furukawa Electric), and an EMCCD camera (iXON+ DU-897; Andor Technology). We used a 4f imaging system composed of two achromatic lenses whose focal positions were 70 and 200 mm, respectively, as a relay optics. The actual magnification of the microscope containing a relay optics was ∼170×. Experiments were carried out in a solution containing 25 mM KCl, 20 mM Hepes (pH 7.5), 1 mM MgCl_2_, 0.2 mM CaCl_2_, 143 mM 2-mercaptoethanol, 12 μM CaM, and 1 mM Trolox in the presence of indicated concentration of ATP. For the assay with single actin filaments, flow chambers were prepared by using no. 1.5 glass coverslips and glass slides (Matsunami Glass). Alpha-actinin (catalog no.: A9776; SIGMA) was used to immobilize F-actin, and casein from bovine milk (07319-82; Nakarai Tesque) was used for glass-surface blocking. The movement of fluorescent HM19 single molecules was monitored and recorded using Aodor SOLIS 32 bit software (version 4.28.30050.0). The experiments were conducted at 22 °C.

### Data analysis

The video images of single molecules captured with a TIRF microscope were analyzed by using in-house 2D Gaussian fitting and tracking software (63). A function for the recorded stage drift was calculated and subtracted from the raw data of each runs for each movie. Run length and velocity of HM19 were concurrently determined by measuring the length and time between on and off the tracks. Time-lapse images, kymographs, and trajectory of the HM19 movements were analyzed using ImageJ software (National Institutes of Health). Numeric calculation was done with Excel software (Microsoft), and the data plotting and statistical analysis were done with Prism software (GraphPad Software, Inc). For step size analysis, an in-house step-fitting software based on an algorithm described in the study by Kerssemakers *et al.* (64) was used as described previously (33, 34).

### 3D deconvolution microscopy

The colocalization of mitochondria membrane vesicles with cellular actin was observed with DeltaVision OMX (GE Healthcare). U2OS cells were cultured on round cover glass (CG15NH, Ø12 mm; Thorlabs), treated with 100 ng/ml nocodazole for 8 h, and demembraned as shown previously in the presence of Alexa Fluor 488 phalloidin, followed by the addition of MitoTracker-stained HMF. After three washes with PBS, the cover glass was mounted to a slide glass using Fluoro gel with DABCO (EM Science) and sealed with nail polish. The fluorescence image was captured with conventional light path setting using DeltaVision OMX software, and the 3D deconvolution and image registration were then applied using SoftWoRx 6.5.2 software (GE). The maximum intensity of a series of *z*-axis images was projected to one *x*–*y* image, and the images were processed using ImageJ software.

### Confocal light microscopy

For the colocalization of HM19 and actin, Halo-HM19-transfected HeLa cells cultured on 3.5 cm glass bottom dishes were fixed with 4% paraformaldehyde for 15 min at 37 °C. After being washed with PBS twice, the cells were treated with 0.1% Triton X-100 in PBS for 3 min. The cells were washed twice with PBS and quenched with 0.1 M glycine in PBS for 15 min at room temperature. Actin and HaloTag proteins were stained for 1 h with Alexa Fluor 647 Phalloidin (0.4 U/ml) and 1 μM HaloTag tetramethylrhodamine ligand, respectively. Unbound fluorophores were removed using five washes with PBS. For the colocalization of HM19 and mitochondria, HeLa cells were transfected with EGFP-HM19s and stained with MitoTracker as described previously. The cells were observed by using an Olympus FV1000 laser scanning confocal microscope system with an IX81 microscope, an objective lens (PLANAPO N 60×, 1.40 NA, oil), 473 nm, 559 nm, and 635 nm lasers (Olympus). The fluorescence signal was captured in a range of 490 to 540 (or 590) nm for EGFP and R110, 575 to 620 nm for tetramethylrhodamine, 575 to 675 nm for MitoTracker, and 655 to 755 nm for Alexa Fluor 647. FV10-ASW Viewer, version 3.1, software was used to control the equipment and data analysis.

## Data availability

All data are contained within the article.

## Supporting information

This article contains supporting information: Fig. S1–S13 and Movies S1–S5.

## Conflict of interest

The authors declare that they have no conflicts of interest with the contents of this article.
