# Supplementary material for: Mitochondria-associated myosin 19 processively transports mitochondria on actin tracks in living cells
Source: J Biol Chem. 2022 Mar 31;298(5):101883. doi: 10.1016/j.jbc.2022.101883 (PMC9065997; doi:10.1016/j.jbc.2022.101883)
Supplement: Supplemental Figures S1–S13 [file mmc6.pdf]

Mitochondria-associated myosin 19 processively transports mitochondria on actin tracks in living cells

Osamu Sato, Tsuyoshi Sakai, Young-yeon Choo, Reiko Ikebe, Tomonobu M. Watanabe, and Mitsuo Ikebe*

*Correspondence Author: Mitsuo Ikebe.
Email: Mitsuo.Ikebe@uthct.edu

This Supplemental information includes:

Figures S1–S13 with the legends
Legends to Movies S1–S5

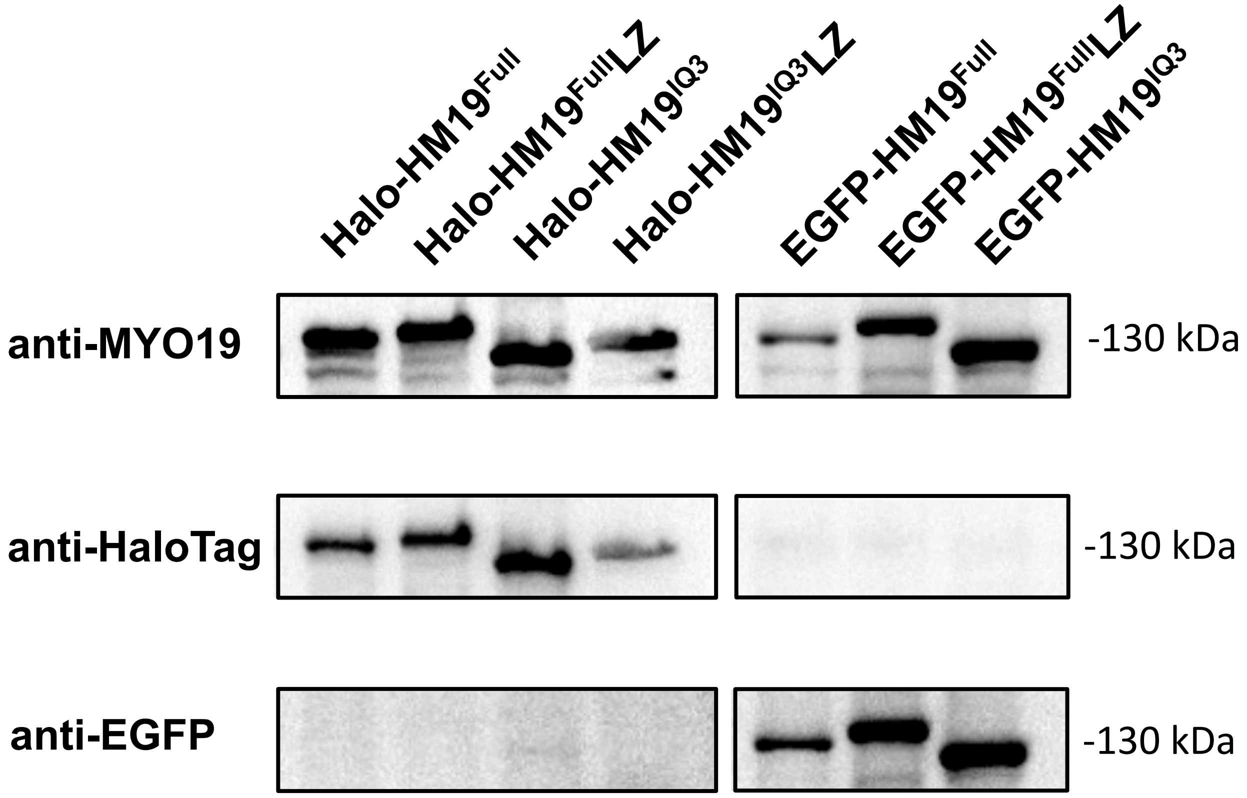

Figure S1. Western blot of Halo-HM19 and EGFP-HM19 proteins used in this study. COS7 cells were transfected with Halo-HM19 and EGFP-HM19 constructs and Western blotting was performed using anti-MYO19, anti-HaloTag, and anti-EGFP antibodies (See Experimental Procedures). The right side of the panels shows the position of 130 kDa protein marker as a molecular mass standard. The estimated molecular masses of HM19 heavy chains according to the position of protein markers were similar to the expected sizes.

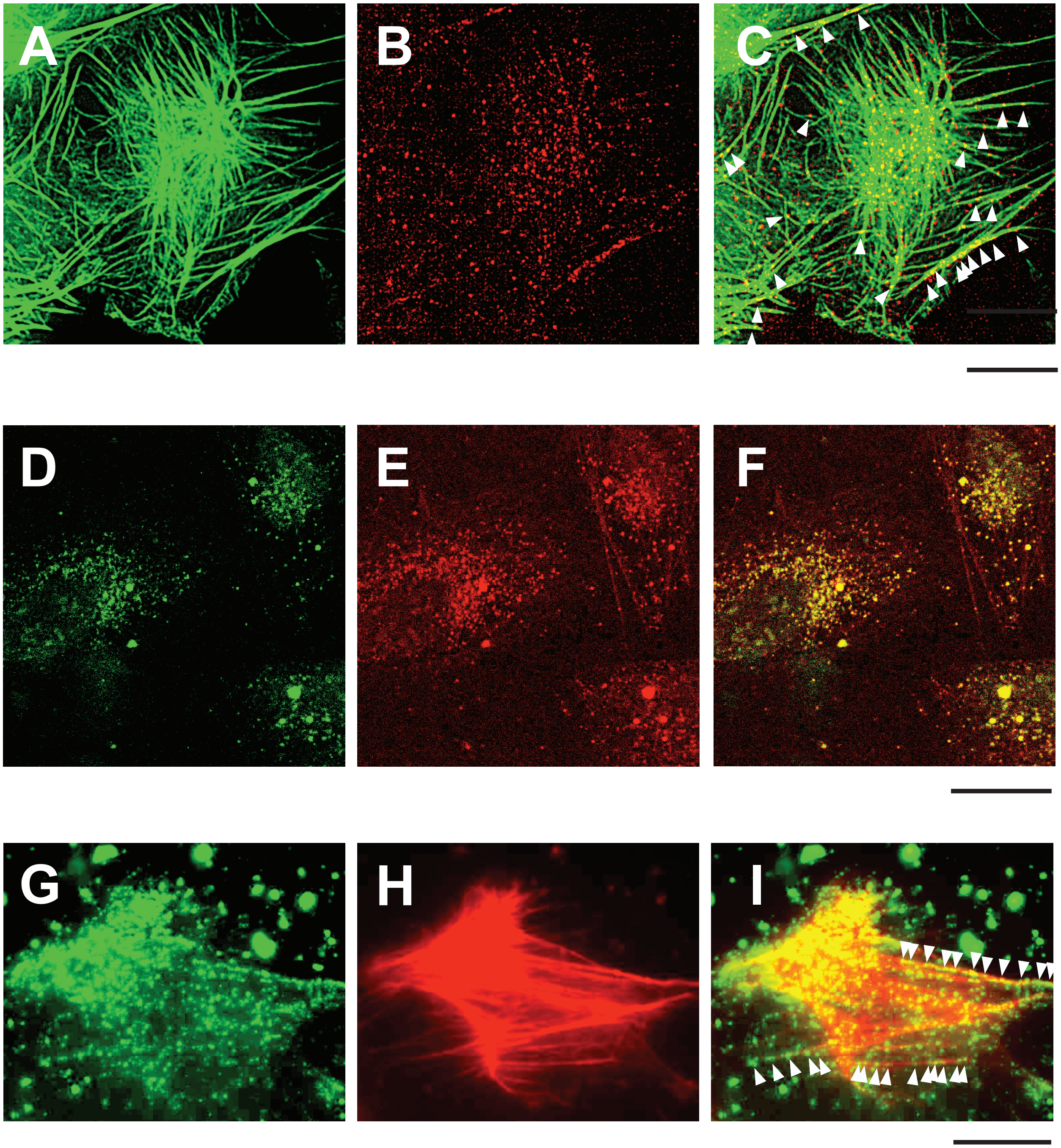
A
B
C
D
E
F
G
H
I

Figure S2. Association of mitochondria vesicles with actin in de-membraned cells. Heavy mitochondrial fraction (HMF) was prepared from HEK293T cells and stained with MitoTracker as described in "Experimental Procedures." De-membraned U2OS cells were stained with Alexa Fluor 488 Phalloidin followed by addition of MitoTracker-stained HMF in the absence of ATP, and the colocalization was observed by 3D deconvolution microscopy (*A*–*C*). (*A*, green) actin, (*B,* red) MitoTracker-stained HMF, (*C*) The merged image of (*A*) and (*B*). Arrowheads show notable localization of HMF on actin tracks. (*D*–*F*) Co-localization of R110-Halo-HM19^{Full}LZ with mitochondria vesicles in de-membraned U2OS cells observed with a confocal microscope. Cultured HEK293T cells were transfected with Halo-HM19^{Full}LZ and stained with R110 Direct HaloTag ligands. The cells were fixed, de-membraned and then mitochondria prepared from R110-Halo-HM19^{Full}LZ-transfected cells stained with MitoTracker were added and observed with a confocal microscope in the absence of ATP. (*D,* green) Halo-HM19^{Full}LZ stained with R110 Direct HaloTag ligands. (*E,* red) Mitochondria vesicles stained with MitoTracker. (*F*) The merged image of (*D*) and (*E*). (*G*–*I*) Localization of mitochondria vesicles prepared from R110-Halo-HM19^{Full}LZ-transfected cells in de-membraned U2OS cells observed with a TIRF microscope. (*G,* green) Halo-M19^{Full}LZ stained by R110 Direct HaloTag ligands. (*H,* red) Cellular actin stained by Alexa Fluor 568 Phalloidin**.** (*I*) The merged image of (*G*) and (*H*). R110-Halo-HM19^{Full}LZ-associated mitochondria vesicles showed notable localization on actin tracks (arrowheads). Bars are 10 μm.

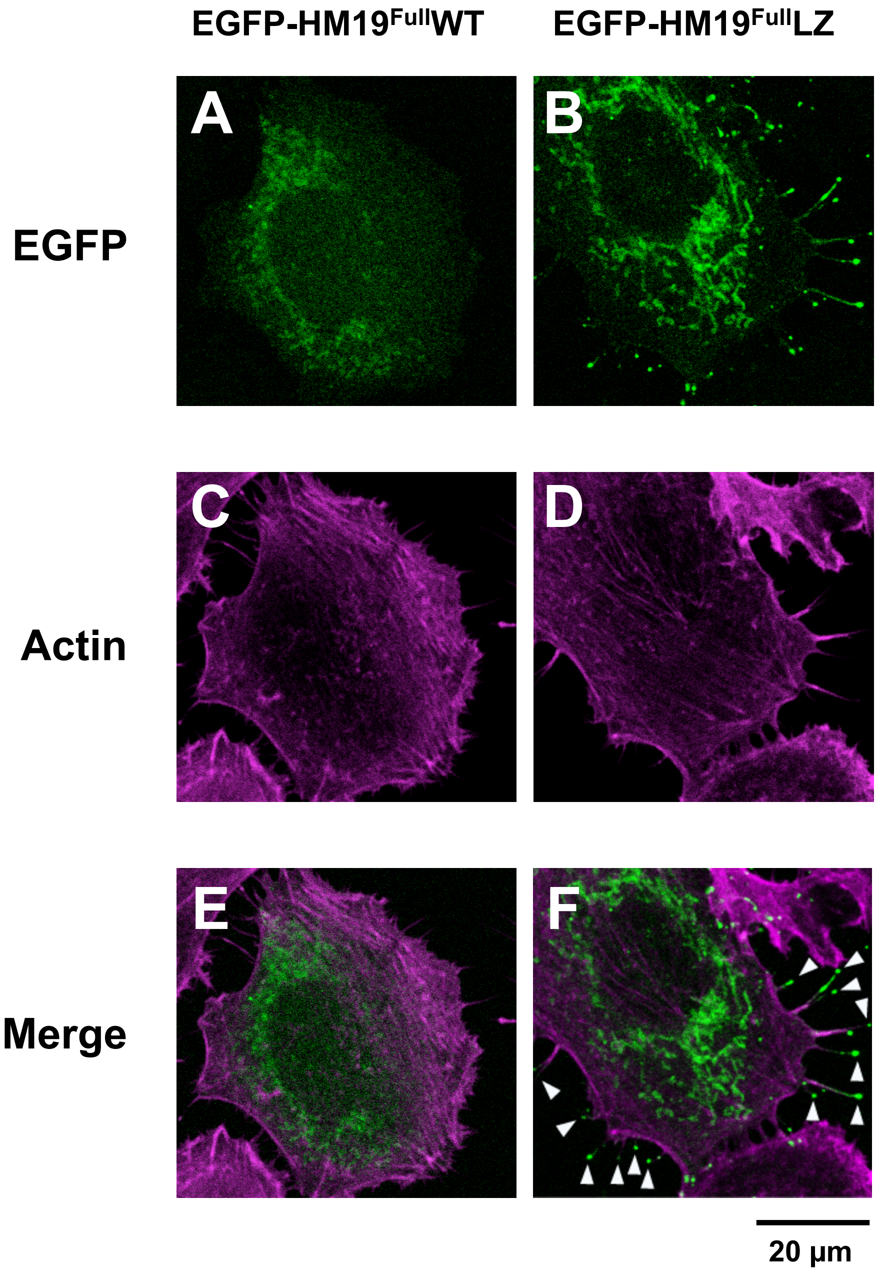

Figure S3. Localizations of EGFP-HM19^{Full}WT and EGFP-HM19^{Full}LZ in HeLa cells. HeLa cells were transfected with EGFP-HM19^{Full}WT (left) and EGFP-HM19^{Full}LZ (right), fixed, permeabilized, stained with Alexa Fluor 647 Phalloidin, and observed with a confocal microscope. EGFP (*A*, *B*) was captured with green channel (Ex. 473 nm and Em. 490 – 590 nm) and actin (*C*, *D*) was captured with far-red channel (Ex. 635 nm and Em. 655 – 755 nm). The merged images (*E*, *F*) are also shown. EGFP-HM19^{Full}LZ shows filopodial tip localization (arrowheads) as well as mitochondria localization. Bar = 20 μm.

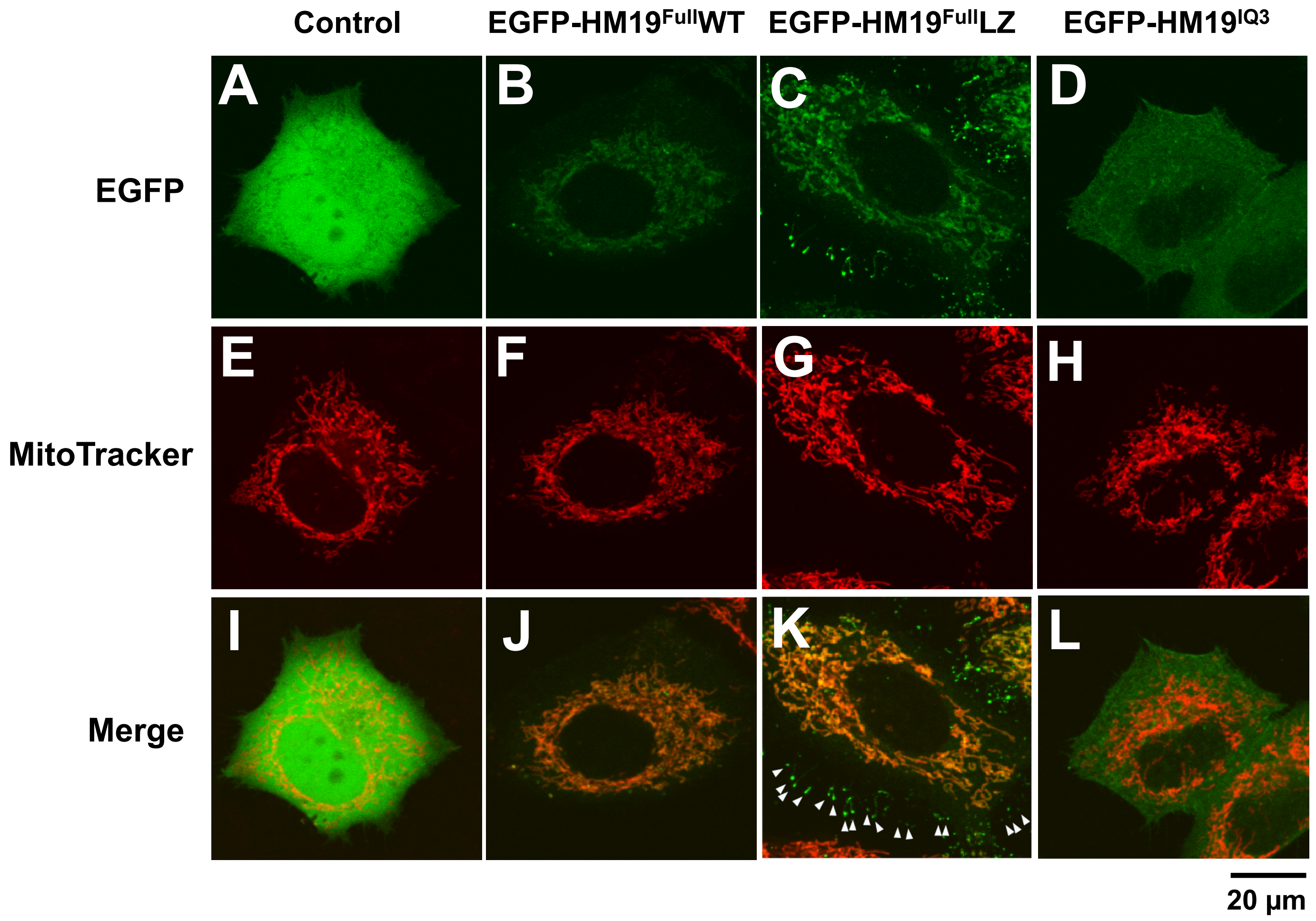

Figure S4. Co-localization of EGFP-HM19 constructs and mitochondria in living HeLa cells. Cultured HeLa cells were transfected with EGFP-HM19 constructs, and the cells were stained with MitoTracker according to "Experimental Procedures," and observed by confocal microscopy. EGFP channel (*A–D*) is shown in green (Ex. 473 nm and Em. 490 – 540 nm), and mitochondria stained by MitoTracker (*E–H*) are shown in Red (Ex. 559 nm and Em. 575 – 675 nm). The merged images at the bottom panels (*I–L*) show the overlay of EGFP channel and MitoTracker channel. Note that the tail-truncated HM19 does not co-localize either with mitochondria nor filopodial tips. Arrowheads show the filopodial tip localization of HM19-forced dimer construct. Control is EGFP expressing cell. Bar = 20 μm.

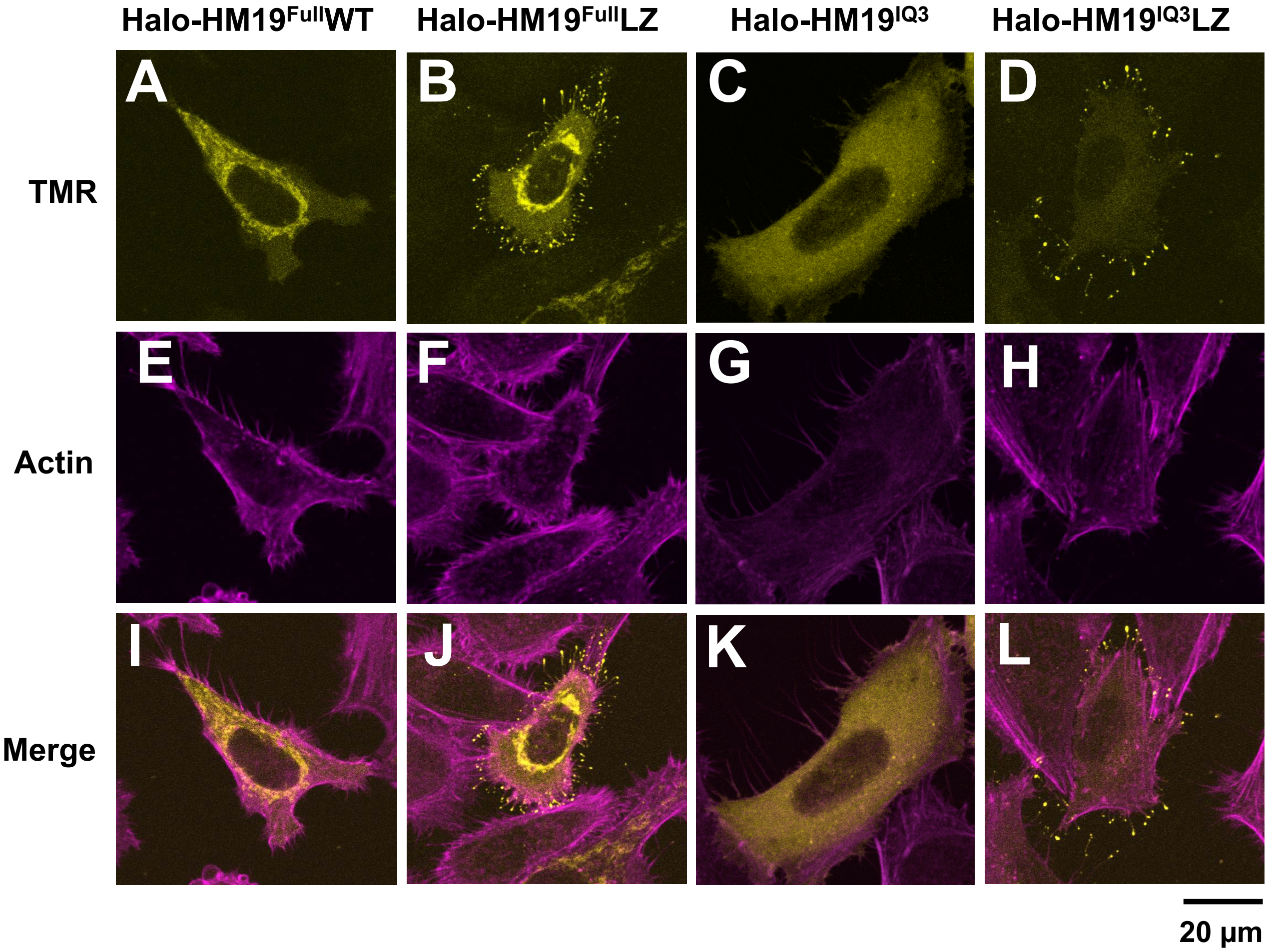

Figure S5. Filopodial tip localization of Halo-HM19 constructs in HeLa cells. Cultured HeLa cells were transfected with Halo-HM19^{Full}WT, Halo-HM19^{Full}LZ, Halo-HM19^{IQ3}, and Halo-HM19^{IQ3}LZ, respectively, and the fixed cells were stained with TMR HaloTag ligands according to "Experimental Procedures." The HM19 constructs stained by TMR (*A–D*) are shown in yellow (Ex. 559 nm and Em. 575 –620 nm), and actin stained by Alexa Fluor 647 phalloidin (*E–H*) is shown in magenta (Ex. 635 nm and Em. 655 – 755nm). The merged images are shown at the bottom panels (*I–L*). Bar = 20 μm.

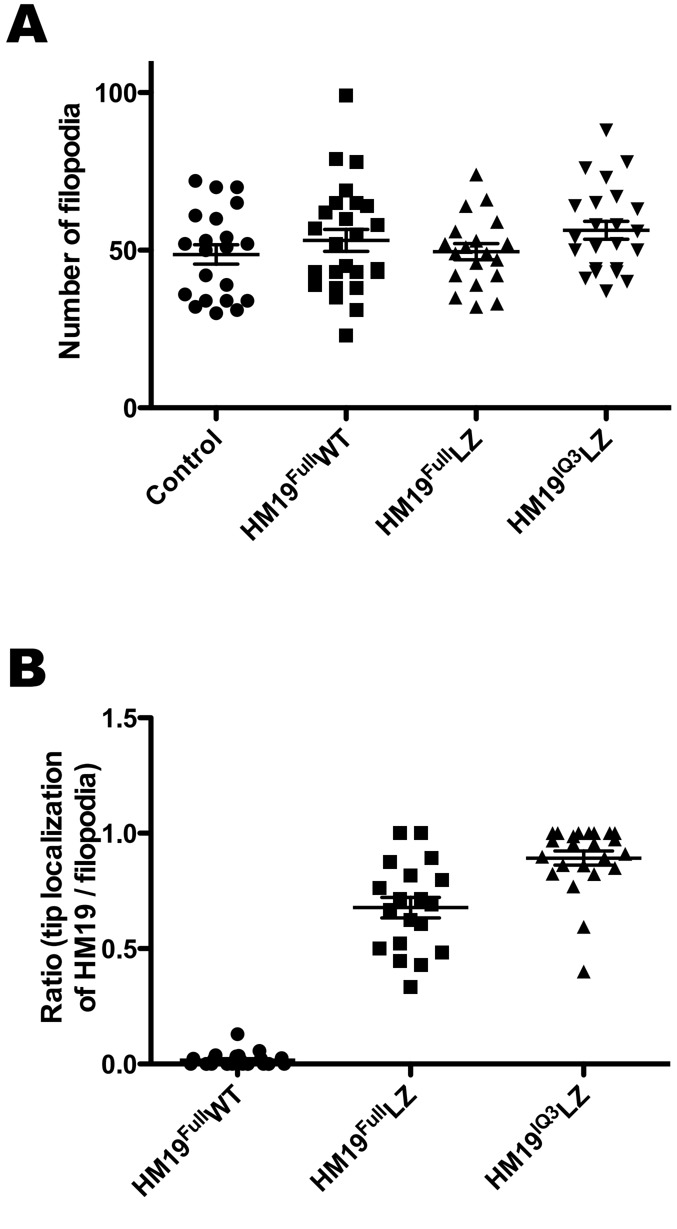

Figure S6. Comparison of filopodial tip localization in Halo-HM19 constructs. HeLa cells were transfected with Halo-HM19^{Full}WT, Halo-HM19^{Full}LZ and Halo-HM19^{IQ3}LZ, fixed, and observed with a confocal microscope as shown in Figure S5. (*A*) The number of filopodia per cell. There was no significant difference between any two groups in one way ANOVA. (*B*) The ratio of filopodial tip localization of HM19 constructs per filopodia. The mean ratios were 0.017 ± 0.006 (mean ± s.e.m., n = 25), 0.68 ± 0.04 (n = 19), and 0.89 ± 0.03 (n = 23) for Halo-HM19^{Full}WT-, Halo-HM19^{Full}LZ-, and Halo-HM19^{IQ3}LZ-transfected cells, respectively. The difference between any two groups was significant at P<0.0001 in one way ANOVA.

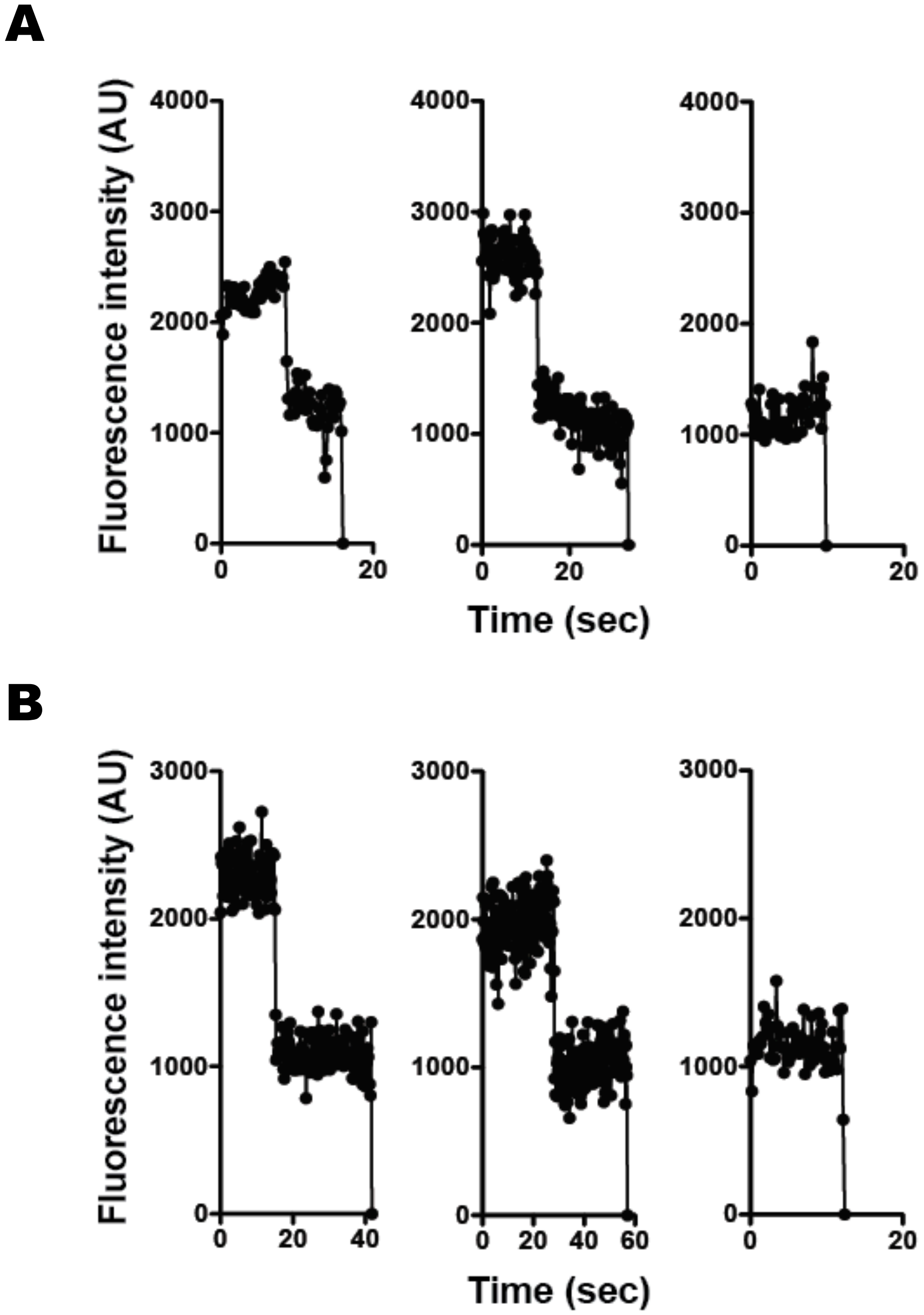

Figure S7. Step-wise photo-bleaching of single molecules of Halo-HM19-forced dimer constructs in living HeLa cells. Halo-HM19^{Full}LZ (*A*) and Halo-HM19^{IQ3}LZ (*B*) were overexpressed in HeLa cells, labeled with R110, and observed with a TIRF microscope by HILO illumination as shown in Fig. 5. The integrated fluorescence intensity of each single spot was analyzed using 2D Gaussian fitting and tracking software, and plotted over time. Representative step-wise photobleaching of R110 fluorescence is shown. The images were taken at 5 fps. AU, arbitrary unit.

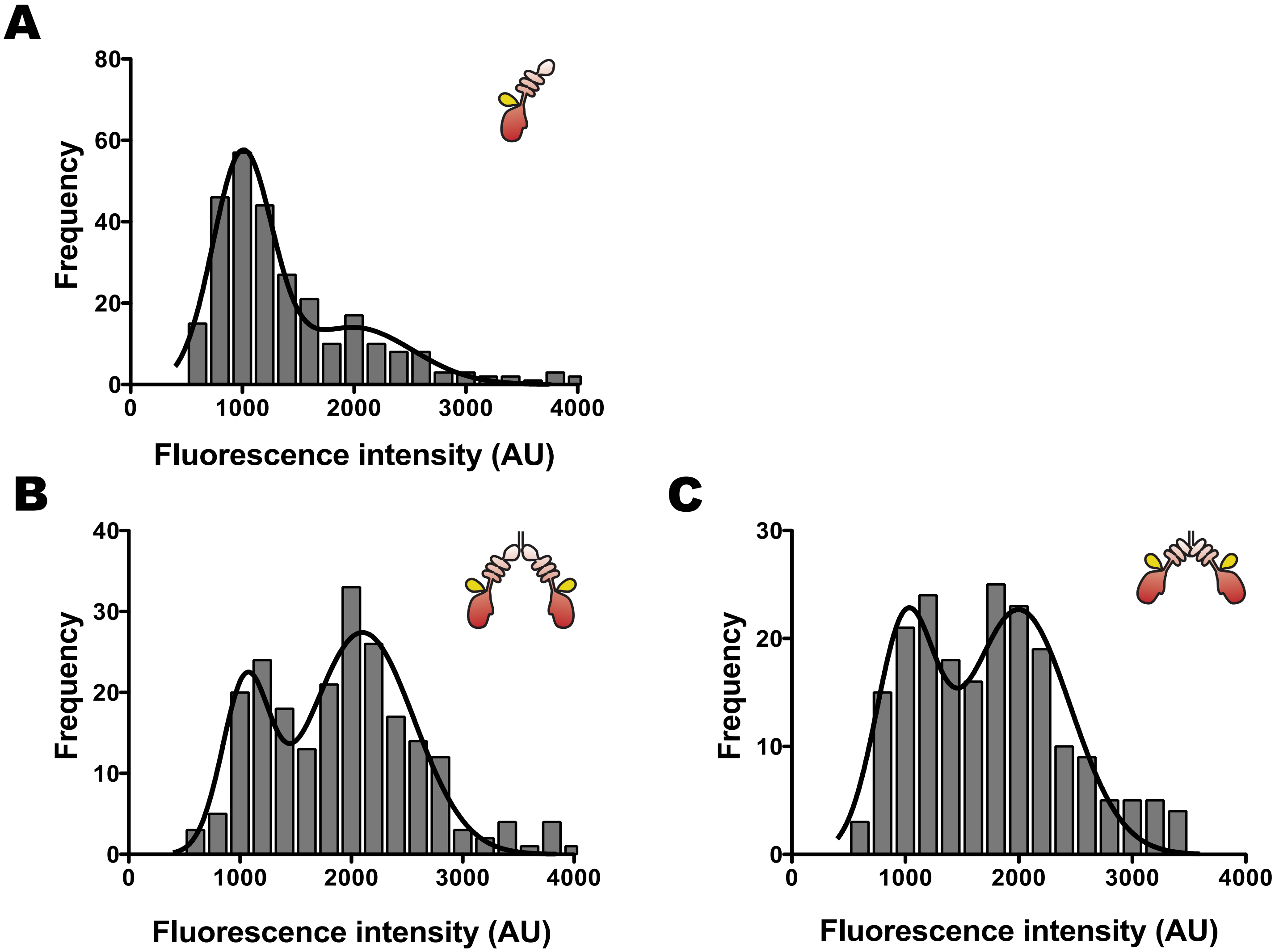

Figure S8. Single particle fluorescence intensities of Halo-HM19-single molecules in living HeLa cells. The fluorescent intensity of each single spot in the first frame was analyzed as shown in Fig. S7. The histograms of fluorescence intensity are shown. (*A*) Halo-HM19^{Full}WT; (*B*) Halo-HM19^{Full}LZ and (*C*) Halo-HM19^{IQ3}LZ. The mean intensities were 1440 ± 45.3 (AU, mean ± s.e.m., n = 282) for R110-Halo-HM19^{Full}WT, 1978 ± 52.0 (AU, n = 226) for R110-Halo-HM19^{Full}LZ, and 1750 ± 46.7 (AU, n = 202) for R110-Halo-HM19^{IQ3}LZ, respectively. Solid lines show the distributions fitted with the two Gaussian equations. Distribution of fluorescence intensities of the particles showed two peaks. The intensity of the first peak was approximately a half of that of the second peak, suggesting that the first and the second peaks represent one and two fluorophores, respectively. AU is arbitrary unit.

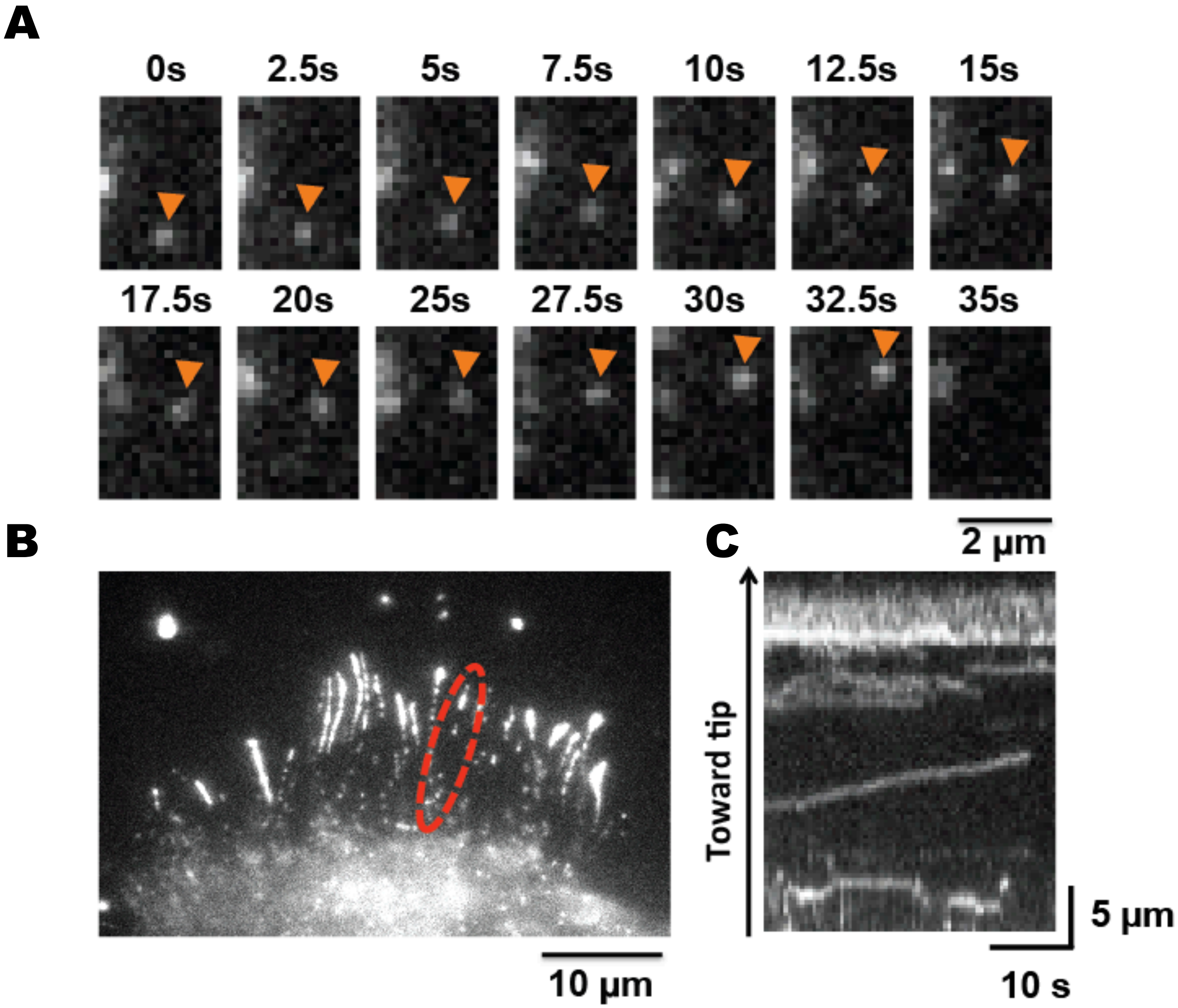

Figure S9. Single-molecule imaging of Halo-HM19^{IQ3}LZ in living cells. Cultured HeLa cells were transfected with Halo-M19^{IQ3}LZ and labeled with R110 Direct HaloTag ligands according to "Experimental Procedures." The movement of R110-Halo-M19^{IQ3}LZ molecules in filopodia was observed at 2 fps with a TIRF microscope by HILO illumination. (*A*) Time-lapse images of R110-Halo-M19^{IQ3}LZ movement in living HeLa cells. Arrowheads indicate the moving R110-Halo-HM19^{IQ3}LZ. Bar, 2 μm. (*B*) Representative image of living HeLa cell. Broken line shows a typical filopodia with R110-Halo-HM19^{IQ3}LZ molecules used for the kymograph analysis shown in (*C*). Bar, 10 μm. (*C*) Representative kymograph showing continuous movement of R110-Halo-HM19^{IQ3}LZ in filopodia. The x axis shows the time and y axis shows the length of the movement.

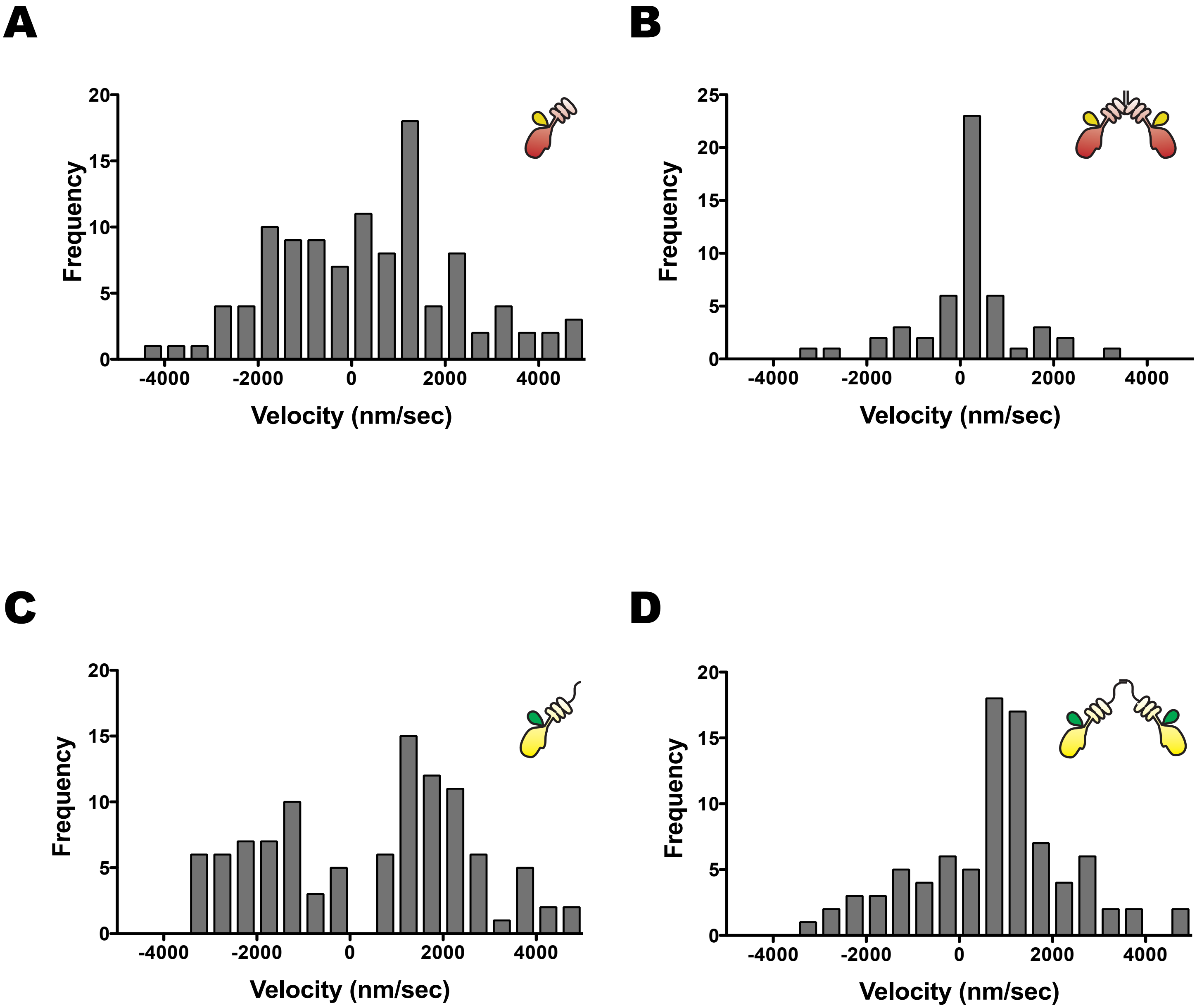

Figure S10. Velocity distribution of single molecules of HM19^{IQ3}, HM19^{IQ3}LZ, and truncated Myo10 constructs monitored with a high frame rate in living HeLa cells. The movements of R110-Halo-HM19^{IQ3} (*A*), R110-Halo-HM19^{IQ3}LZ (*B*), EGFP-BM10^{IQ3} SAH (*C*), and EGFP-BM10^{IQ3} SAH CC (*D*) in filopodia of live HeLa cells were observed at 10 fps with a TIRF microscope by HILO illumination. The velocity of each moving fluorescent spot was determined from the kymograph. The distribution of the velocity in each fluorescent spot is shown (n=109 for R110-Halo-HM19^{IQ3}, n=52 for R110-Halo-HM19^{IQ3}LZ, n=110 for EGFP-BM10^{IQ3} SAH and n=110 for EGFP-BM10^{IQ3} SAH CC). BM10, Bovine Myo10; SAH, Stable single α-helix; and CC, coiled-coil.

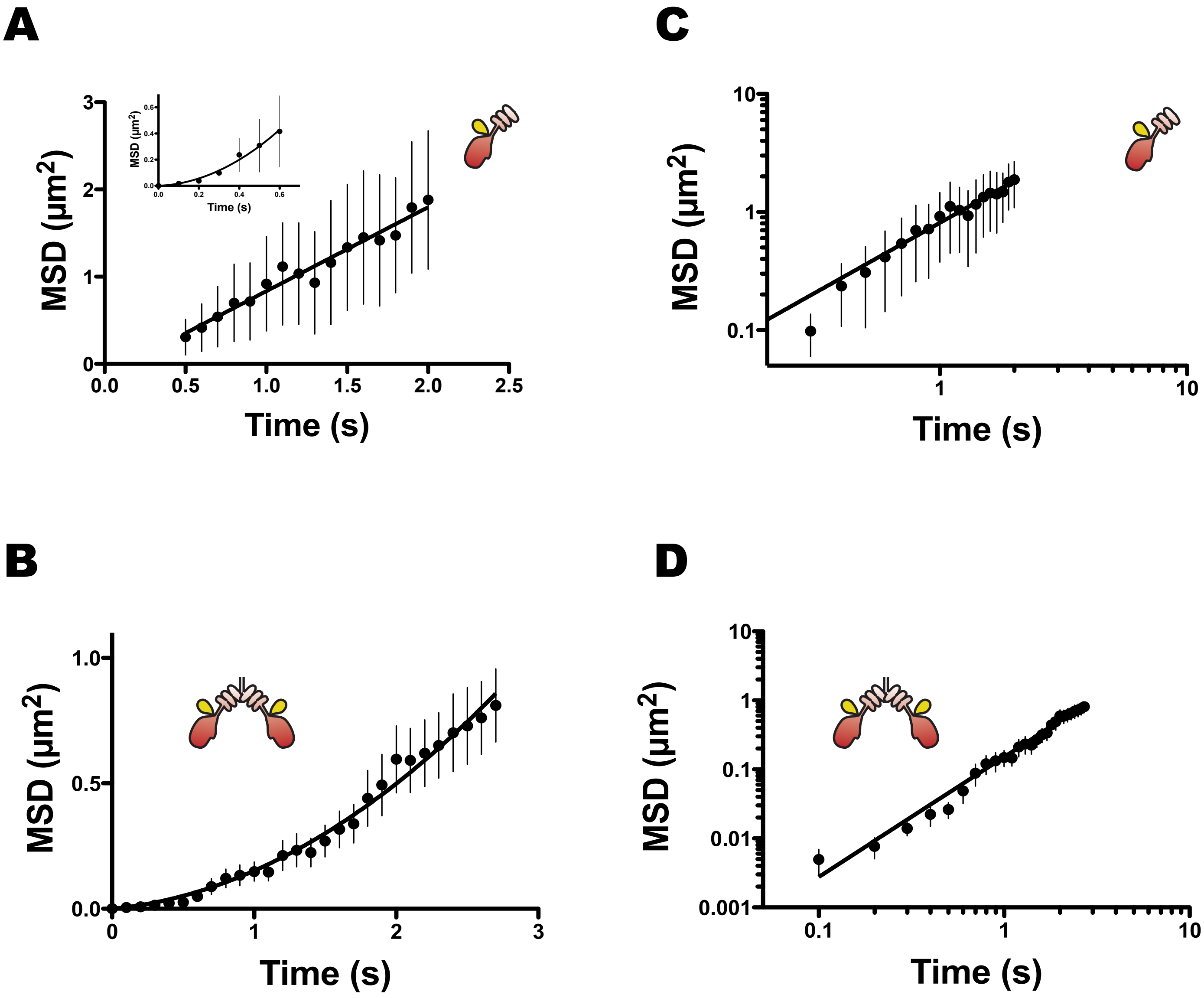

Figure S11. Mean square displacement (MSD) analysis of HM19^{IQ3} and HM19^{IQ3}LZ movements in filopodia of living HeLa cells. The movement of R110-Halo-HM19^{IQ3} (*A*, *C*), R110-Halo-HM19^{IQ3}LZ (*B, D*) in filopodia of live HeLa cells were observed at 10 fps as shown in Fig. S10. The moving fluorescent spots were tracked with a 2D Gaussian fitting and tracking software, and the MSD was calculated. (*A*) MSD plot for R110-Halo-HM19^{IQ3}. The plot is fitted with two equations, $f(t) = 4Dt + c$ for 0.5 – 2.0 s ($D = 0.24 \pm 0.01$ $\mu m^2/s$), and $f(t) = 4Dt + v^2t^2$ for 0–0.6 s (inset, solid line, $v = 1.0 \pm 0.5$ $\mu m/s$, $D = 0.02 \pm 0.14$ $\mu m^2/s$). (*B*) MSD plot for R110-Halo-HM19^{IQ3}LZ. The plot is fitted with an equation of $f(t) = 4Dt + v^2t^2$ for 0 – 2.7 s (solid line, $v = 0.31 \pm 0.03$ $\mu m/s$, $D = 0.014 \pm 0.010$ $\mu m^2/s$), where v = velocity; t = time; and D = diffusion coefficient. (*C, D*) Log-log plots fitted with $f(t) = ct^{\alpha}$, where c = constant, t = time, and α = slope of log-log plots. The slope (α) for R110-Halo-HM19^{IQ3} (*C*) and R110-Halo-HM19^{IQ3}LZ (*D*) are calculated to be 1.17 ± 0.35 and 1.74 ± 0.17, respectively. Error bars are s.e.m. (n = 6).

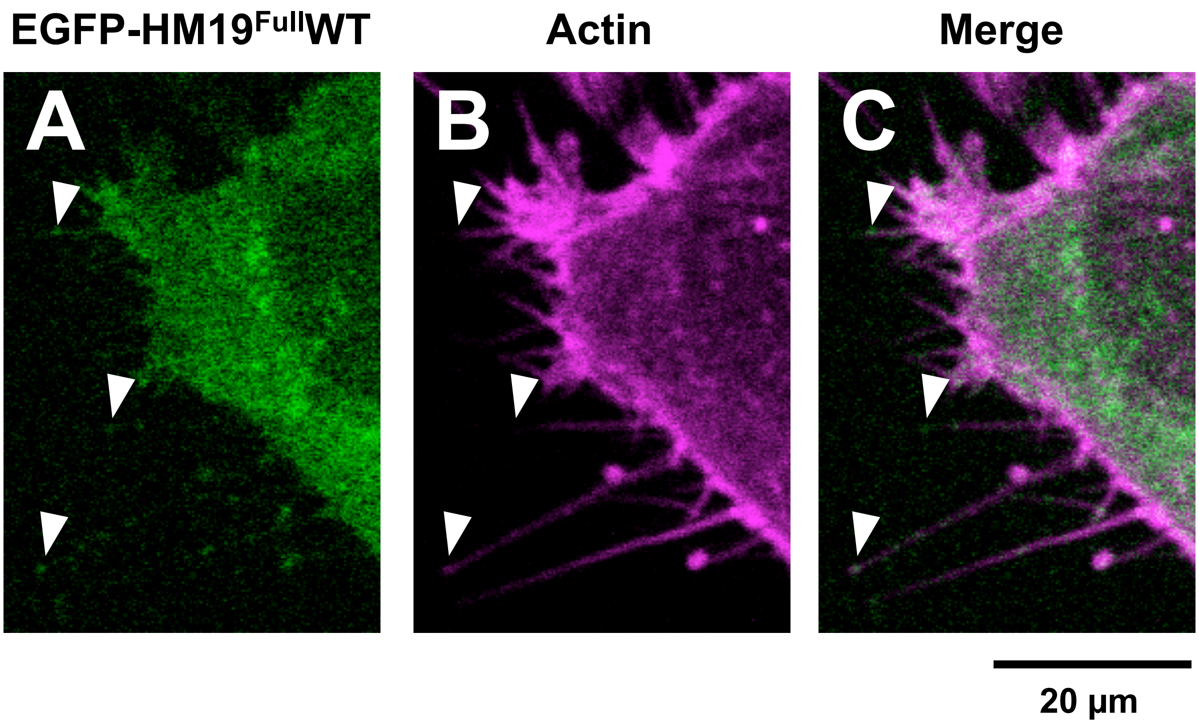

Figure S12. Filopodial tip localization of EGFP-HM19^{Full}WT in HeLa cells. EGFP-HM19^{Full}WT-transfected HeLa cells were observed with a confocal microscope. Unlike HM19^{IQ3}, we can find HM19^{Full}WT localized at the tip of some filopodia. (*A*) EGFP-HM19^{Full}WT; (*B*) Actin; (*C*) The merged image of (*A*) and (*B*). Arrowheads indicate EGFP-HM19^{Full}WT localized at filopodial tips. Bar = 20 μm.

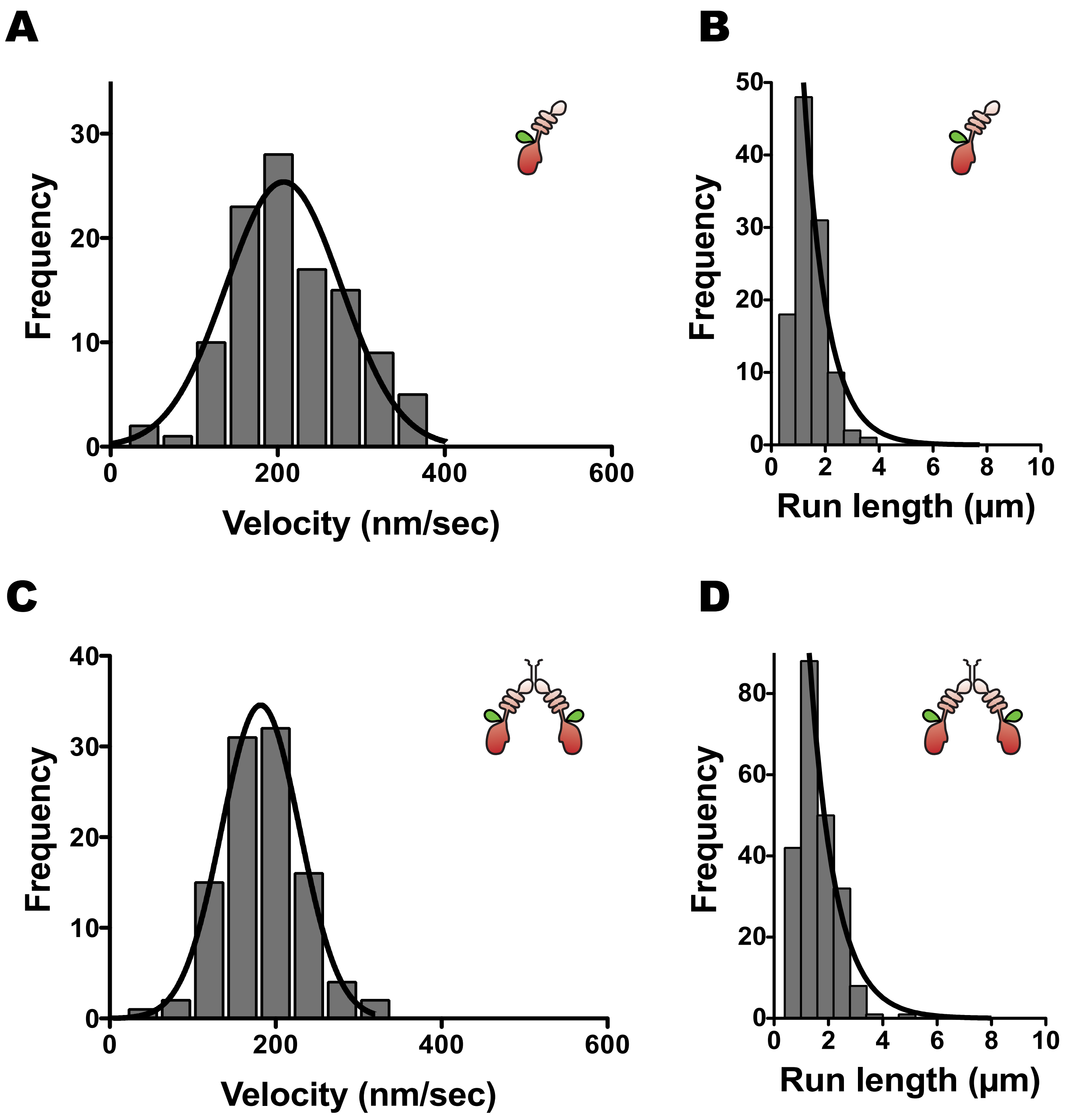

Figure S13. Velocities and run lengths of EGFP-HM19^{Full}WT and EGFP-HM19^{Full}LZ in living HeLa cells. The movements of EGFP-HM19^{Full}WT and EGFP-HM19^{Full}LZ in filopodia in HeLa cells were observed with a TIRF microscope by HILO illumination. The distributions of the velocity (*A, C*) and the run length (*B, D*) of EGFP-HM19^{Full}WT (*A, B*) and EGFP-HM19^{Full}LZ (*C, D*) are shown. The mean velocities of EGFP-HM19^{Full}WT and EGFP-HM19^{Full}LZ were 0.216 ± 0.007 μm/s (mean ± s.e.m., n=110) and 0.185 ± 0.005 μm/s (mean ± s.e.m., n=103), respectively. Solid lines in (*A*) and (*C*) show Gaussian distributions. Solid lines in (*B*) and (*D*) show the best fit to a single exponential equation, $R_0 e^{-r/\lambda}$ (See Fig. 2). The average run lengths of EGFP-HM19^{Full}WT and EGFP-HM19^{Full}LZ were 0.85 ± 0.14 μm (mean ± s.e.m., n=110) and 0.94 ± 0.09 μm (mean ± s.e.m., n=222), respectively.

Legends to Movies S1–S5

Movie S1. Movement of HMF obtained from HM19^{Full}LZ-overexpressed cells on de-membraned U2OS cells. HMF obtained from Halo-HM19^{Full}LZ-overexpressed cells was stained with R110 Direct HaloTag ligands. The movement of HMF in 1 mM ATP was captured at 5 fps. The trajectory of the light spots was analyzed using TrackMate (plug-in software of Image J) and the image was inserted at the end of the movie. The movie replay speed is edited as 2X using Adobe Premiere software. The actual time line is shown at the bottom of the movie.

Movie S2. Movement of HM19^{Full}LZ on de-membraned U2OS cells. The movement of purified EGFP-HM19^{Full}LZ on de-membraned U2OS cells in 1 mM ATP was captured at 10 fps. The trajectory of the light spots was shown at the end of the movie. 2X replay speed.

Movie S3. Movement of HM19^{Full}WT single molecules on filopodia in a living HeLa cell monitored with a low frame rate. Cultured HeLa cells were transfected with Halo-HM19^{Full}WT and labeled with R110 Direct HaloTag ligands. The movement of R110-Halo-HM10^{Full}WT in filopodia was then monitored at 2 fps. 2X replay speed.

Movie S4. Movement of HM19^{Full}WT single molecules in filopodia of a living HeLa cell monitored with a high frame rate. Cultured HeLa cells were transfected with Halo-HM19^{Full}WT and labeled with R110 Direct HaloTag ligands. The movement of R110-Halo-HM10^{Full}WT in filopodia was then monitored at 10 fps. 2X replay speed.

Movie S5. Movement of HM19^{Full}LZ-Qdot on single actin filaments. Purified EGFP-HM19^{Full}LZ was attached to Qdot525 and the movement on single actin filaments at 2 μM ATP were then monitored at 10 fps using TIRF microscope. 1X replay speed. The trajectory of the light spots analyzed by TrackMate is shown in the second movie.
